# UHRF1 inhibition epigenetically reprograms cancer stem cells to suppress the tumorigenic phenotype of hepatocellular carcinoma

**DOI:** 10.1038/s41419-023-05895-w

**Published:** 2023-06-28

**Authors:** Yanchen Wang, Pengchao Hu, Fenfen Wang, Shaoyan Xi, Shasha Wu, Liangzhan Sun, Yuyang Du, Jingyi Zheng, Hui Yang, Mao Tang, Han Gao, Hao Luo, Yue Lv, Jingsong Yan, Xijun Ou, Yan Li

**Affiliations:** 1 Harbin Institute of Technology, Harbin, China; 2 Department of Biology, School of Life Sciences, Southern University of Science and Technology, Shenzhen, China; 3 Department of Pathology, Sun Yat-Sen University Cancer Center, Guangzhou, China; 4 State Key Laboratory of Oncology in South China and Collaborative Innovation Center for Cancer Medicine, Sun Yat-sen University Cancer Center, Guangzhou, China

**Keywords:** Cancer stem cells, Liver cancer, DNA methylation

## Abstract

Cancer stem cells (CSCs) contribute to tumor initiation, progression, and recurrence in many types of cancer, including hepatocellular carcinoma (HCC). Epigenetic reprogramming of CSCs has emerged as a promising strategy for inducing the transition from malignancy to benignity. Ubiquitin-like with PHD and ring finger domains 1 (UHRF1) is required for DNA methylation inheritance. Here, we investigated the role and mechanism of UHRF1 in regulating CSC properties and evaluated the impact of UHRF1 targeting on HCC. Hepatocyte-specific *Uhrf1* knockout *(Uhrf1*^*HKO*^*)* strongly suppressed tumor initiation and CSC self-renewal in both diethylnitrosamine (DEN)/CCl_4_-induced and *Myc*-transgenic HCC mouse models. Ablation of UHRF1 in human HCC cell lines yielded consistent phenotypes. Integrated RNA-seq and whole genome bisulfite sequencing revealed widespread hypomethylation induced by UHRF1 silencing epigenetically reprogrammed cancer cells toward differentiation and tumor suppression. Mechanistically, UHRF1 deficiency upregulated CEBPA and subsequently inhibited GLI1 and Hedgehog signaling. Administration of hinokitiol, a potential UHRF1 inhibitor, significantly reduced tumor growth and CSC phenotypes in mice with *Myc*-driven HCC. Of pathophysiological significance, the expression levels of UHRF1, GLI1, and key axis proteins consistently increased in the livers of mice and patients with HCC. These findings highlight the regulatory mechanism of UHRF1 in liver CSCs and have important implications for the development of therapeutic strategies for HCC.

## Introduction

Hepatocellular carcinoma (HCC) accounts for 75–85% of primary liver cancer cases [1]. High HCC-associated mortality is partly due to the high proportion of patients diagnosed with advanced-stage HCC and the lack of effective treatments [2]. Therefore, new treatment strategies are urgently needed.

Cancer stem cell (CSC) or tumor-initiating cell models suggest that a subpopulation of stem-like cells within tumors, which have self-renewal and differentiation characteristics, are responsible for tumor initiation, treatment resistance, and recurrence. These rare cells have been reported in various cancer types, including HCC, and several CSC markers, such as CD44 and CD133, have been identified [3, 4]. Liver CSCs exhibit frequent activation of the Wnt/β-catenin, Hedgehog, and Notch signaling pathways, which play important roles in liver development, hepatic growth, and liver CSC self-renewal [5–7]. Moreover, stemness-related transcription factors such as SOX2 and c-MYC are aberrantly expressed in liver cancer [8]. Similar to normal tissue stem cells, CSCs with high malignancy can be converted into differentiated cells with low tumorigenicity [9]. Such plasticity of CSCs can be exploited by differentiation therapy to deplete CSC subpopulations and to eradicate cancer.

Available evidence suggests that DNA methylation reprogramming is a key epigenetic mechanism that plays a vital role in CSC plasticity [10, 11]. De novo DNA methylation is induced by the DNA methyltransferases DNMT3a and DNMT3b. During cell division, ubiquitin-like with PHD and ring finger domains 1 (UHRF1) recruits DNA methyltransferase 1 (DNMT1) to hemi-methylated DNA sites and maintains DNA methylation, through which daughter cells inherit DNA methylation patterns [12]. Previous studies have reported that ablation of UHRF1 leads to the differentiation of different adult stem cells [13–16]. Recently, Hu et al. reported that targeting UHRF1 could eradicate leukemia-initiating cells in myeloid leukemia, revealing the role of UHRF1 in maintaining cancer initiating cell [17]. UHRF1 is frequently overexpressed in several types of cancers and plays an oncogenic role in cancer progression [18, 19]. However, the functional role and mechanism of UHRF1 in regulating CSC properties in the liver remain unknown. Although there have been several studies regarding UHRF1 in liver injury and cancer, most of the results were obtained from HCC cells in vitro [18]. Regarding the in vivo data generated from model organisms, some findings appear inconsistent or even contradictory. For example, UHRF1 overexpression in zebrafish hepatocytes causes DNA hypomethylation by destabilizing and delocalizing Dnmt1 [20], whereas Magnani et al. found that deletion of *uhrf1* also led to DNA methylation loss in zebrafish livers [21]. Moreover, DNA hypomethylation caused by *uhrf1* loss induced the activation of transposable elements and interferon response in zebrafish, but the transposable elements were suppressed through the redistribution of H3K27me3 in *Uhrf1* deletion mice [22]. Therefore, it is necessary to depict the methylome upon UHRF1 depletion at base resolution and investigate the resulting transcriptional reprogramming. Furthermore, in vivo models and data are required to determine whether UHRF1 could be a therapeutic target for HCC.

Herein, we demonstrated that UHRF1 functions as an epigenetic regulator that reprograms CSCs toward differentiation and tumor suppression via GLI1/Hedgehog and Wnt signaling. In addition, UHRF1 ablation via genetic knockout or pharmacological inhibition alleviates hepatocarcinogenesis and CSC phenotypes in mice. Our findings provide mechanistic insights and identify UHRF1 as a potential target for liver cancer therapies.

## Materials and methods

### Clinical samples

Primary HCC specimens and paired nontumor tissues were collected with informed consent from patients who underwent hepatectomy at Sun Yat-sen University Cancer Center (Guangzhou, China). Seventy-five pairs of the frozen primary tumor and adjacent nontumor tissues (cohort 1) were used for reverse transcription quantitative PCR (RT-qPCR) analysis of mRNA expression, and eight pairs were used for western blotting (WB) analysis of protein expression. A tissue microarray (TMA) containing 177 primary HCC tumor tissues (cohort 2) was used for the immunohistochemical detection of protein expression. The clinical specimens used in this study were approved by the Committee for Ethical Review of Research Involving Human Subjects at Sun Yat-sen University Cancer Center.

### Animal experiments

All animal experiments were reviewed and approved by the Institutional Animal Care and Use Committee of Southern University of Science and Technology. All mice had a C57bl/6 background. Hepatocyte-specific *Uhrf1* knockout (*Uhrf1*^*HKO*^) mice were obtained by crossing *Uhrf1*^*flox/flox*^ mice (Shanghai Model Organisms Center) with albumin-Cre (*Alb-Cre*) mice (Shanghai Model Organisms Center). The resulting *Uhrf1*^*flox/flox*^*Alb-Cre* mice served as the experimental group (*n* = 6), and *Uhrf1*^*flox/flox*^ mice served as the control group (*n* = 5). Only male mice were used in this study.

To establish a fibrosis-associated HCC model, intraperitoneal injection of 20 mg/kg DEN (Sigma, #N0258-1g) was administered within two weeks of birth to initialize the HCC process. Then, tetrachloride (CCl_4_) (5 µl/g body weight, diluted with olive oil) was intraperitoneally injected twice a week after an interval of 6 weeks to promote HCC progression. Tissue was harvested from two groups of mice. The first group consisted of 5 control mice and 6 *Uhrf1*^*HKO*^mice sacrificed at 7 months, which was used to evaluate the effect of UHRF1 knockout on HCC carcinogenesis. The second group consisted of 3 control mice and 3 *Uhrf1*^*HKO*^ mice sacrificed at 4, 5, 6, and 7 months. This cohort was used to track UHRF1 expression at different stages of HCC carcinogenesis. The liver tissues were collected for subsequent experiments.

To establish the *Myc*-driven HCC model, hepatocyte-specific *Myc* knock-in mice (*Myc*^*HKI/+*^) were generated by crossing *Hipp11-stop*^*flox/flox*^*-Myc* mice (Shanghai Model Organisms Center) with albumin-Cre mice. Liver-specific *Uhrf1* knockout mice (*Uhrf1*^*HKO*^*Myc*^*HKI/+*^) were generated by hybridizing *Uhrf1*^*flox/flox*^*Alb*^*Cre/+*^ and *Uhrf1*^*flox/flox*^*Myc*^*HKI/+*^ mice. *Uhrf1*^*HKO*^*Myc*^*HKI/+*^ mice served as the experimental group (*n* = 6), and *Myc*^*HKI/+*^ mice served as the control group (*n* = 6). The mice were sacrificed at 9 weeks of age, and liver tissues were collected for subsequent experiments.

For hinokitiol treatment, *Myc*^*HKI/+*^ mice were intraperitoneally injected with vehicle (control group, *n* = 6) or 25 mg/kg hinokitiol (treatment group, *n* = 8) twice a week from 4 to 9 weeks of birth. The mice were sacrificed at 9 weeks of age, and liver tissues were collected for subsequent experiments.

### Statistics

Statistical analyses were performed using SPSS and GraphPad Prism. An unpaired Student’s *t* test was used to examine differences between any two preselected groups. Differences in survival were analyzed using Kaplan–Meier curves and log-rank tests. Correlations between two statistical variables were analyzed using Spearman’s correlation analysis. The results are expressed as the mean ± standard error of the mean. *P* < 0.05 was considered to be statistically significant.

For further details regarding the materials and methods used, please refer to the Supplementary Information.

## Results

### Hepatocyte-specific *Uhrf1* knockout alleviates DEN/CCl_4_-induced hepatocarcinogenesis in vivo

**Fig. 1:**
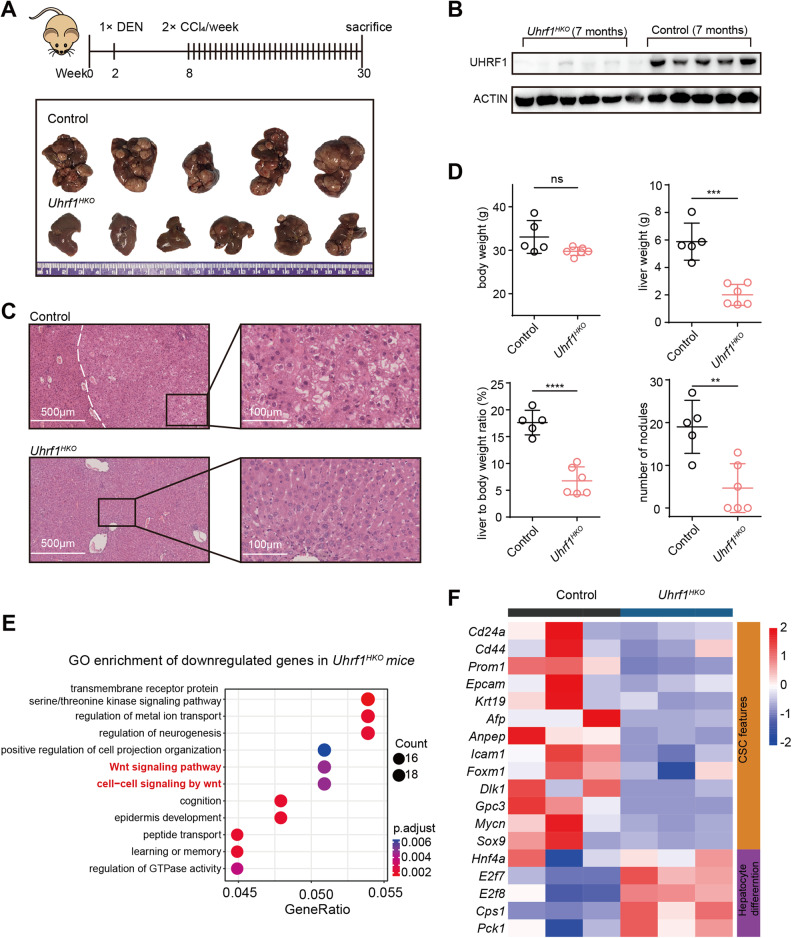
Hepatocyte-specific *Uhrf1* knockout alleviates DEN/CCl_4_-induced hepatocarcinogenesis in vivo. **A** Schematic summary of the DEN/CCl_4_-induced mouse model of HCC (top). Image of mouse liver tissues from the indicated groups (bottom). **B** Immunoblotting analysis of the indicated proteins in liver tissues of *Uhrf1*^*HKO*^ mice compared with control mice. **C** Representative H&E staining of liver tissue from mice at 30 weeks (White dotted line indicates the boundary between tumor and nontumor). **D** Body weight, liver weight, liver to body weight ratio, and number of nodules from the indicated groups were measured. **E** Gene Ontology analysis of downregulated genes in *Uhrf1*^*HKO*^ mice compared with control mice. **F** Heatmap displaying stemness- and differentiation-related genes that were differentially expressed between *Uhrf1*^*HKO*^ and control mice. The color of each cell shows the Z score (log2 of relative abundance scaled by SD) of the mRNA in that sample. Mean ± SD. *P* values were determined using unpaired Student’s *t* test. ^∗∗^*P* < 0.01, ^∗∗∗^*P* < 0.001, ^∗∗∗∗^*P* < 0.0001. Ns not significant.

To investigate the effects of UHRF1 loss-of-function on HCC development and progression, we generated hepatocyte-specific *Uhrf1* knockout mice (*Uhrf1*^*HKO*^) by crossing *Uhrf1*^*flox/flox*^ mice with albumin-Cre mice. *Uhrf1*^*HKO*^ mice developed normally into viable adults, with no difference from age-matched control mice in terms of body weight, gross appearance of the liver, or histologically assessed hepatic architecture. Fibrosis-associated mouse hepatocarcinogenesis was induced by a single injection of DEN followed by repeated administration of CCl_4_, with 100% of the mice developing liver tumors at 7 months of age (Fig. 1A and S1A). In *Uhrf1*^*HKO*^ livers, both *Uhrf1* mRNA expression (Fig. S1B) and protein levels (Fig. 1B) were dramatically reduced, demonstrating the efficacy of this knockout strategy. *Uhrf1* knockout strongly reduced tumor incidence and volume, whereas the livers of control mice were occupied by multiple tumors (Fig. 1A and S1A). Hematoxylin and eosin (H&E) staining confirmed the histological architecture of the corresponding liver tissues (Fig. 1C). A robust decrease in tumor numbers, liver weight, and liver to body weight ratios was detected in *Uhrf1*^*HKO*^ mice, three of which did not form tumors (Fig. 1D). Transcriptomic analysis of DEN/CCl_4_-induced HCC showed that 958 genes were downregulated following *Uhrf1* knockout (Fig. S1C), among which the Wnt signaling pathway was enriched (Fig. 1E and S1E). A total of 551 genes were upregulated and enriched in molecular catabolic and metabolic processes (Fig. S1C and D). Moreover, UHRF1 deletion reduced the expression of known CSC signature genes (*Cd24*, *Cd44*, *Cd133*, *Epcam*, *Krt19*, *Afp*, *Anpep*, *Icam1*, *Foxm1*, *Dlk1*, *Gpc3*, *Mycn*, *Sox9*, and *Hnf4a*) (Fig. 1F), among which FOXM1 was reported to promote UHRF1 expression [23]. In contrast, UHRF1 depletion enhanced the expression of genes associated with hepatocyte differentiation (*E2f7*, *E2f8*, *Cps1*, and *Pck1*) (Fig. 1F). These in vivo results suggested that *UHRF1* knockout strongly attenuated hepatocarcinogenesis, possibly by regulating the self-renewal and differentiation of liver CSCs.

### UHRF1 is required for the maintenance of the CSC phenotype

**Fig. 2:**
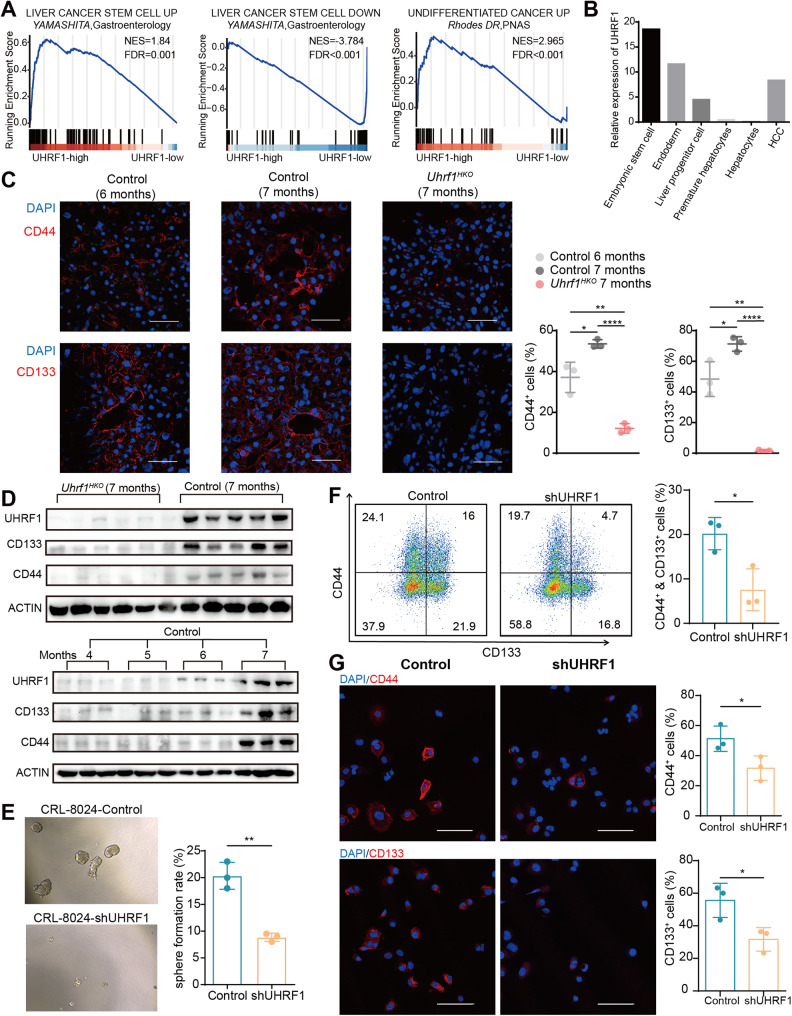
UHRF1 silencing abolishes the CSC phenotype of HCC cells. **A** Gene set enrichment analysis of cancer stem cell-associated gene sets in *UHRF1* high versus low patients from the TCGA-LIHC dataset. **B** The mRNA expression of *UHRF1* at different hepatic developmental stages and in HCC tumor tissues. **C** Immunofluorescence analysis of CD44 and CD133 expression in liver tissues at the indicated time points. The nuclei were stained with DAPI (blue). Scale bar: 100 μm (*n* = 3). **D** Immunoblotting analysis of the indicated proteins in liver tissues of mice at different time points. **E** Representative images and quantification of spheroids formed by the indicated stable cell lines (*n* = 3). **F** The CSC subpopulations were evaluated by flow cytometry. CRL-8024 cells were stained with anti-CD133-PE and anti-CD44-APC antibodies. The percentage of CD44^+^CD133^+^ cells was calculated and depicted in the bar chart (*n* = 3). **G** Immunofluorescence analysis of CD44 and CD133 expression in CRL-8024 cells. The nuclei were stained with DAPI (blue). Scale bar: 100 μm. Mean ± SD. *P* values were determined using unpaired Student’s *t* test. ^∗^*P* < 0.05, ^∗∗^*P* < 0.01, ^∗∗∗^*P* < 0.001, ^∗∗∗∗^*P* < 0.0001.

Previous studies have identified gene expression signatures of stemness and differentiation in cancer. Yamashita et al. revealed a gene cluster upregulated in hepatic stem cell-like HCCs and a downregulated gene cluster associated with mature hepatocyte function. Rhodes et al. characterized a transcriptional profile that is commonly activated in various types of undifferentiated cancer [24, 25]. Gene set enrichment analysis revealed that the above two stemness-associated gene sets were positively enriched in tumors with high *UHRF1* mRNA expression, whereas the differentiation-associated gene set was negatively enriched in *UHRF1*-high patients from the TCGA-LIHC dataset (Fig. 2A). In addition, the expression of *UHRF1* was positively correlated with activation of the canonical Hedgehog and Wnt signaling pathways (Fig. S2A). We previously established an in vitro hepatocyte differentiation model [26] and found that UHRF1 showed an oncofetal-like gene expression pattern during liver development and tumorigenesis (Fig. 2B). These findings suggest that UHRF1 modulates the stemness of tumor-initiating cells. Supporting this notion, the expression level of UHRF1 gradually increased during HCC pathogenesis in the mouse model, accompanied by elevated protein levels of CD133 and CD44, whereas *Uhrf1* deletion almost completely abrogated this elevation (Fig. 2C, D, and S2B). To further investigate whether UHRF1 affects human liver CSC attributes, we established stable UHRF1 knockdown or overexpression in SNU-449 and CRL-8024 HCC cell lines (Fig. S2C and S3A). Ectopic overexpression of UHRF1 did not affect tumor cell proliferation or migration, which may have resulted from functional redundancy (Fig. S3B–D). In contrast, UHRF1 silencing significantly reduced tumorigenicity and migration (Fig. S2D–F). The sphere formation assay showed that UHRF1 silencing significantly reduced the number of spheroids formed by stem cells (Fig. 2E). Flow cytometry confirmed a lower proportion of CD44^+^CD133^+^ liver CSCs in UHRF1 knockdown cells than in control cells (Fig. 2F). Consistently, immunofluorescence signals for CD133 and CD44 were significantly decreased after UHRF1 depletion (Fig. 2G). In general, these results suggested a pivotal role for UHRF1 in CSC maintenance.

### UHRF1 knockdown induces genome-wide loss of DNA methylation and modulates gene transcription

**Fig. 3:**
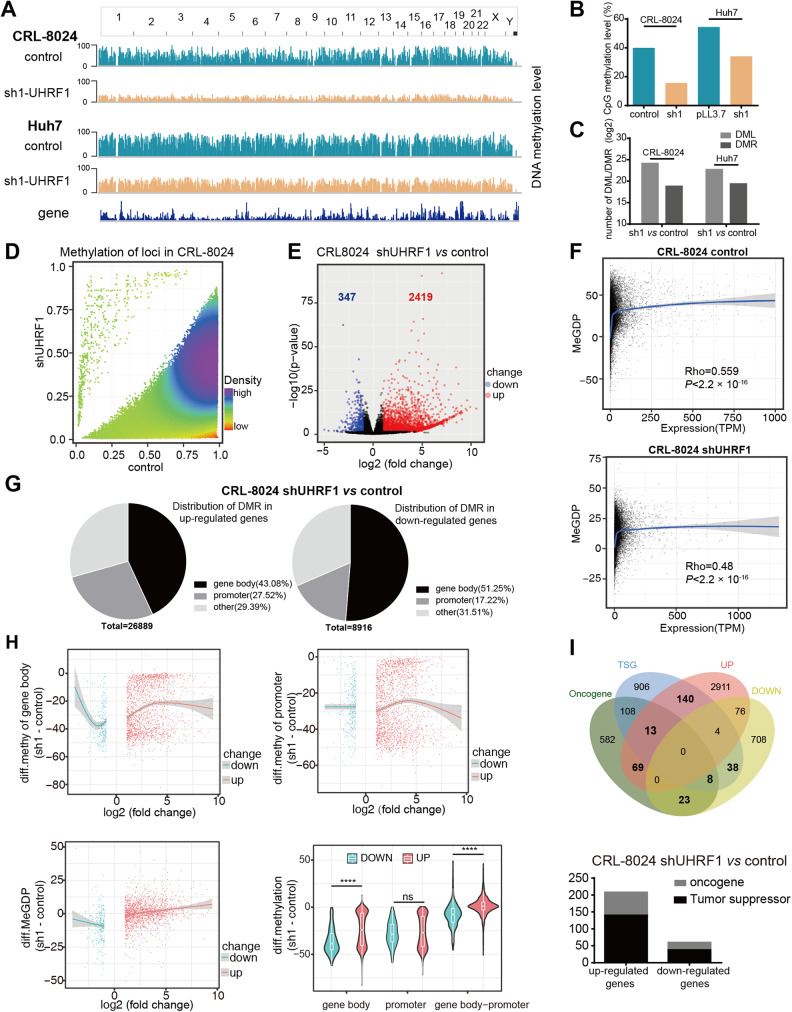
UHRF1 knockdown induces genome-wide loss of DNA methylation and modulates gene transcription. **A** The distribution of methylation levels in the whole genome. **B** The methylation level of CpG sites in the indicated cells. **C** Number of DML (differentially methylated locus) and DMR (differentially methylated region) in the indicated cells. **D** Density scatter plot of DNA sites with different methylation levels after UHRF1 knockdown. **E** Volcano plot for differentially expressed genes between control and UHRF1 knockdown CRL-8024 cells. **F** Scatter plot showing the correlation between DNA methylation and gene expression (TPM). MeGDP: numeral methylation difference between gene body and promoter. **G** Distribution of DMR of upregulated genes and downregulated genes. **H** Scatter plot showing the correlation between DNA methylation changes and gene expression changes (log2 (fold change)) after UHRF1 depletion in CRL-8024 cells. The values of DNA methylation change are depicted in the violin plot. The *y*-axis represents the numeral methylation difference in the gene body, promoter and MeGDP between the UHRF1 knockdown group and the control groups. **I** Venn diagram among the four datasets in CRL-8024 cells. The number of oncogenes and tumor suppressors that were upregulated or downregulated after UHRF1 knockdown are shown in the bar graph. *P* values using Spearman’s correlation coefficient (**F**) or unpaired Student’s *t* test (**H**). ^∗∗∗∗^*P* < 0.0001. Ns not significant.

To further elucidate the underlying mechanism, RNA-seq, and whole genome bisulfite sequencing were conducted to compare the transcriptome and DNA methylation profiles between UHRF1 knockdown cells and control cells. Two independent shRNAs (sh1 and sh2) targeting UHRF1 generated highly correlated transcriptional patterns (Fig. S4A). We performed the following analysis based on the sh1 dataset due to the higher knockdown efficiency and greater demethylation effects. Genome-wide hypomethylation was observed in both CRL-8024 and Huh7 cells (Fig. 3A–C). These differentially methylated loci (DML) and differentially methylated regions (DMR) were distributed throughout the genome with a similar pattern between CRL-8024 and Huh7 cells (Fig. S4B). Among the DMLs, 99.9% were hypomethylated while less than 0.1% were hypermethylated (Fig. 3D and S4C). The majority of differentially expressed genes (DEGs) were upregulated (Fig. 3E and S4D). We then attempted to delineate how UHRF1-mediated methylation affected gene transcription. DNA methylation at promoter regions was originally described as a ‘silencing’ epigenetic mark, whereas methylation in the gene body is positively correlated with gene expression [27]. A recent study suggested that the methylation difference between the gene body and promoter (MeGDP) is a better predictor of gene expression [28]. Integrative analysis of the methylome and transcriptome confirmed that gene expression was positively correlated with the level of DNA methylation in MeGDP (Fig. 3F and S4E).

More specifically, we compared the distribution of DMRs. The DMRs of upregulated and downregulated genes were mainly located in the promoter and gene body, respectively (Fig. 3G). In addition, the mean change in MeGDP was less than zero among the downregulated genes. In contrast, the average change in MeGDP of upregulated genes was greater than zero, and the degree of MeGDP change was positively correlated with the change in gene expression (Fig. 3H). This finding is consistent with the prevailing model, that is, a higher proportion and greater degree of promoter demethylation tend to contribute to gene upregulation, while a higher proportion and greater degree of gene body demethylation tend to lead to gene downregulation. Moreover, promoter DNA hypermethylation has been shown to result in the inactivation of tumor suppressor genes and is therefore implicated in carcinogenesis [29]. We found that global hypomethylation induced by UHRF1 knockdown upregulated a higher proportion of tumor suppressor genes (TSGs) than oncogenes (Fig. 3I and S4F). The above results suggested that UHRF1 knockdown induced genome-wide loss of DNA methylation, and the intricate DNA methylation balance between the promoter region and gene body region modulated gene transcription.

### Ectopic expression of GLI1 reverses the phenotype caused by UHRF1 silencing

**Fig. 4:**
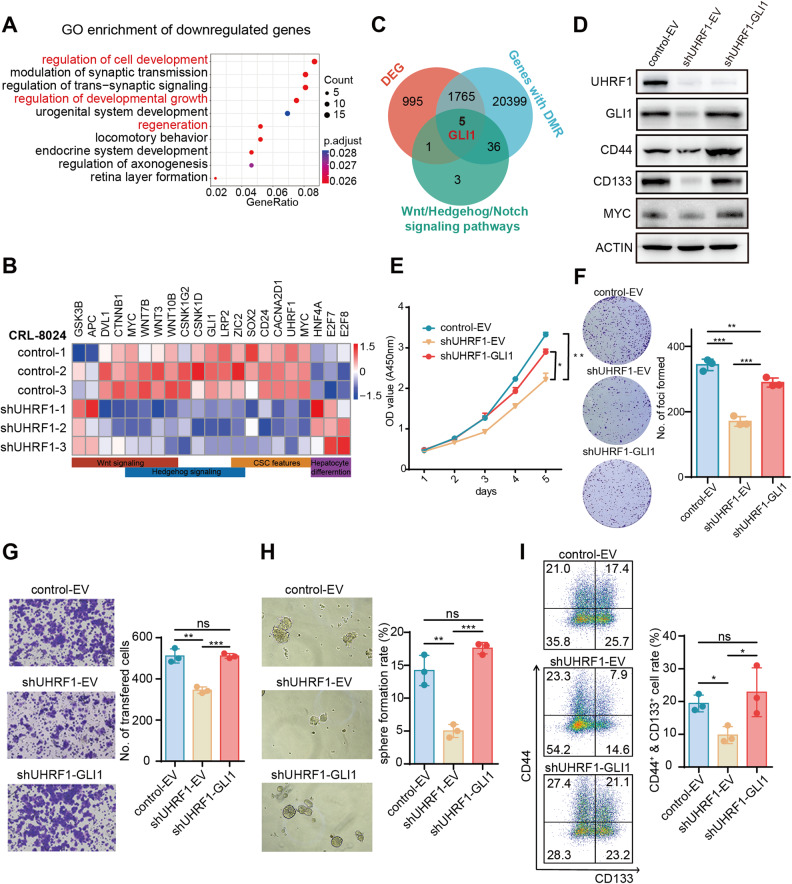
Ectopic expression of GLI1 reverses the phenotype caused by UHRF1 silencing. **A** Gene Ontology analysis of downregulated genes in the CRL-8024 UHRF1 knockdown group compared with the CRL-8024 control group. **B** Heatmap displays stemness- and differentiation-related genes that were differentially expressed between CRL-8024 control and UHRF1 knockdown cells. The color of each cell shows the Z score (log2 of relative abundance scaled by SD) of the mRNA in that sample. **C** Venn diagram among three datasets in CRL-8024 cells. **D** WB analysis of UHRF1, GLI1, CD44, and CD133 protein expression in CRL-8024 cells with UHRF1 knockdown (shUHRF1-EV) and subsequent GLI1 ectopic expression (shUHRF1-GLI1). EV: empty vector. **E** CCK8 assay was used to assess the viability of the indicated stable CRL-8024 cell lines. **F** Representative images and quantification of foci formation induced by the indicated CRL-8024 cells (*n* = 3). **G** Representative images and statistical results of the transwell assay of the indicated CRL-8024 cells (*n* = 3). **H** Representative images and quantification of spheroids formed by the indicated stable CRL-8024 cell lines (*n* = 3). **I** The CSC subpopulations were evaluated by flow cytometry. CRL-8024 cells were stained with anti-CD133-PE and anti-CD44-APC antibodies. The percentages were calculated and depicted in the bar chart (*n* = 3). Mean ± SD. *P* values were determined using unpaired Student’s t test. ^∗^*P* < 0.05, ^∗∗^*P* < 0.01, ^∗∗∗^*P* < 0.001. Ns not significant.

Gene Ontology functional enrichment analysis of the DEGs between control and UHRF1 knockdown cells showed that upregulated genes were enriched in cell junction and extracellular structure organization terms (Fig. S5A and B). The downregulated genes were involved in the regulation of development, growth, and regeneration (Fig. 4A). Similar to *Uhrf1*^*HKO*^ mice, genes downregulated in UHRF1 knockdown cells were associated with known CSC signatures (CD24, SOX2, MYC, CACNA2D1, and ZIC1) as well as Hedgehog/Wnt signaling, and the upregulated genes were associated with hepatocyte differentiation (E2F7, E2F8, and HNF4A) (Fig. 4B). Thus, we attempted to identify key downstream effectors of UHRF1 by overlapping the core components of CSC-related pathways, DEGs, and genes with DMRs. Five genes were identified, including GLI1, which was reported to be regulated by the UHRF1/DNMT1 complex in medulloblastoma [30] (Fig. 4C and S5C). To explore whether UHRF1 affected CSC characteristics via GLI1, we replenished GLI1 in shUHRF1 CRL-8024 cells (Fig. 4D). Ectopic GLI1 expression partially rescued the inhibitory effects of UHRF1 deficiency on cell proliferation and migration (Fig. 4E–G). Importantly, the restoration of GLI1 expression in shUHRF1 cells reversed the suppression of sphere formation (Fig. 4H). The proportion of CD44^+^CD133^+^ liver CSCs (Fig. 4I) and the protein levels of CD44, CD133, and c-MYC (Fig. 4D) recovered to even higher levels than those in the control group. In summary, these results showed that UHRF1 silencing reduced cancer cell stemness by suppressing GLI1 expression. GLI1 ectopic expression largely reversed the inhibition of cell proliferation, migration, and self-renewal induced by UHRF1 knockdown.

### UHRF1 silencing downregulates GLI1 expression through CEBPA

**Fig. 5:**
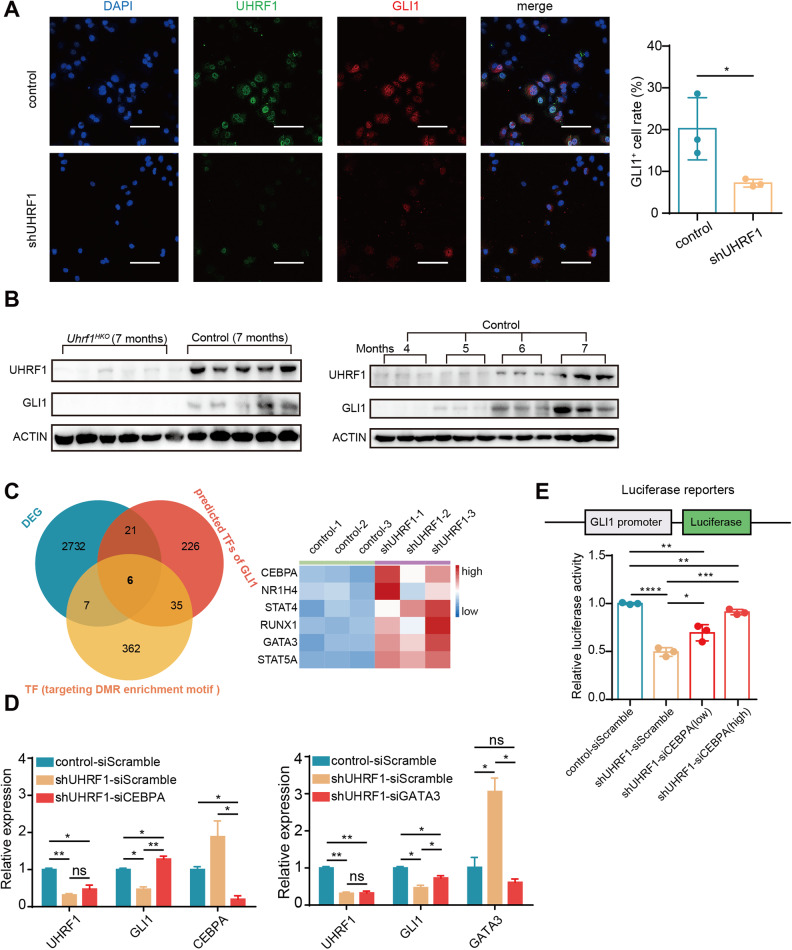
UHRF1 silencing downregulated GLI1 through CEBPA. **A** Immunofluorescence analysis of GLI1 (red) and UHRF1 (green) expression in UHRF1 knockdown and control CRL-8024 cells. The nuclei were stained with DAPI (blue). Scale bar: 100 μm. **B** Protein levels of UHRF1 and GLI1 in liver tissues from *Uhrf1*^*HKO*^ mice and corresponding control mice at different time points in the DEN/CCl_4_ induced HCC model. **C** Venn diagram among three datasets in CRL-8024 cells. The relative expression of overlapping genes is represented in a heatmap. **D** The relative mRNA expression of genes in CRL-8024 cells after CEBPA or GATA3 silencing. **E** The *GLI1* promoter was cloned into the pGL3-basic vector and cotransfected with siCEBPA or scramble siRNA for dual-luciferase assays (low/high: low/high concentration of siRNA) (*n* = 3). Mean ± SD. *P* values were determined using unpaired Student’s *t* test. ^∗^*P* < 0.05, ^∗∗^*P* < 0.01, ^∗∗∗∗^*P* < 0.0001. Ns not significant.

Upon UHRF1 silencing, GLI1 was downregulated at both the mRNA and protein levels (Fig. 4D and S6A). Consistently, the immunofluorescence signal of GLI1 was significantly decreased after UHRF1 knockdown (Fig. 5A). In the DEN/CCl_4_-induced HCC model, UHRF1 expression progressively increased with increasing GLI1 levels. Hepatic-specific knockout of *Uhrf1* inhibited GLI1 expression to barely detectable levels (Fig. 5B and S6B). We then investigated how UHRF1 regulates GLI1 expression.

UHRF1 depletion induced DNA hypomethylation, including the promoter of GLI1 (Fig. S6C). To identify the transcription factors (TFs) that affect GLI1 expression, motif enrichment analysis was performed using DMR sequences flanking the 5’ end of the GLI1 transcription start site. The TFs obtained in this approach further overlapped with the DEGs and TFs of GLI1 predicted from the online database AnimalTFDB 3.0. Six TFs, namely, CEBPA, NR1H4, STAT4, RUNX1, GATA3, and STAT5A, were screened out, and their expression was upregulated in shUHRF1 CRL-8024 cells (Fig. 5C). Specific siRNAs were delivered to silence each of these candidate TFs, and only CEBPA and GATA3 depletion restored GLI1 expression (Fig. 5D and S6D). CEBPA exhibited a greater effect on the transcriptional regulation of GLI1 compared to GATA3. Similarly, the decreased luciferase activity of the *GLI1* promoter (−2000/100 bp) upon UHRF1 knockdown recovered more after CEBPA silencing than GATA3 silencing (Fig. 5E and S6E). Taken together, the present data indicated that UHRF1 deficiency upregulated CEBPA, which subsequently suppressed the transcription of GLI1.

### Genetic knockout or chemical inhibition of UHRF1 attenuates *Myc*-driven HCC development in mice

**Fig. 6:**
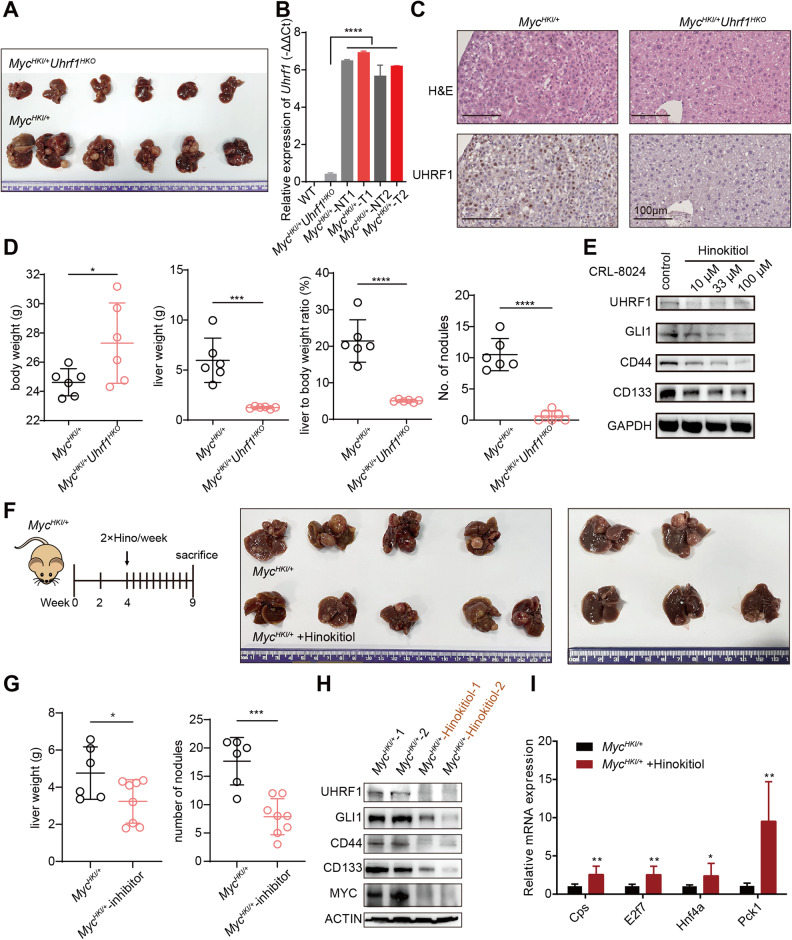
Genetic knockout or chemical inhibition of *Uhrf1* attenuates *Myc*-driven HCC development in mice. **A** Image of mouse liver tissues from the indicated mice 10 weeks after birth. **B** RT-qPCR analysis of *Uhrf1* expression in livers isolated from mice as indicated. WT: wild type, NT: nontumor, T: tumor. **C** Representative H&E staining and UHRF1 staining of liver tissues from the indicated mice. **D** Body weight, liver weight, liver/body weight ratio, and number of nodules from the indicated mice were measured at 10 weeks of birth (*n* = 6 per group). **E** WB analysis of UHRF1, GLI1, CD44, and CD133 expression in CRL-8024 control cells and hinokitiol-treated cells (10–100 μM). **F** Study design (left). Image of mouse liver tissues from *Myc*^*HKI/+*^ mice treated with vehicle (*n* = 6) or hinokitiol (*n* = 8) at 9 weeks of birth (right). **G** Liver weight and number of nodules from the indicated mice were measured at 9 weeks of birth. **H** Detection of UHRF1, GLI1, CD44, and CD133 expression in mouse liver tissues as indicated by WB analysis. **I** Relative mRNA expression of differentiation-associated genes in mouse liver tissues as indicated (*n* = 3). Mean ± SD. *P* values were determined using unpaired Student’s *t* test. ^∗^*P* < 0.05, ^∗∗∗^*P* < 0.001, ^∗∗∗∗^*P* < 0.0001. Ns not significant.

HCC, similar to many other cancers, is highly heterogeneous at the molecular level. We investigated whether targeting UHRF1-controlled CSC self-renewal is an effective therapeutic option for HCC with different molecular etiologies. The DEN/CCl_4_ model is suitable for studying the biology of fibrosis-associated hepatocarcinogenesis. Liver-specific *Myc* oncogene transgenic mice can mimic HCC with c-MYC amplification, which is frequently detected in HCC [31, 32]. Next, we tested the effect of UHRF1 targeting on *Myc*-overexpressing mice. Hepatocyte-specific *Myc* knock-in mice (*Myc*^*HKI/+*^) were generated, and 100% of these mice had HCC within three months of birth (Fig. 6A). Interestingly, *Myc* overexpression strongly induced UHRF1 expression compared to C57bl/6 wild-type mice (Fig. 6B). Successful deletion of *Uhrf1* was detected in hepatocyte-specific *Uhrf1* knockout mice (*Myc*^*HKI/+*^*Uhrf1*^*HKO*^), as confirmed by qPCR and immunohistochemistry (IHC) staining (Fig. 6B, C). UHRF1 deficiency almost completely inhibited *Myc*-driven HCC formation, as demonstrated by the reduced liver weight, liver to body weight ratios, and tumor numbers (Fig. 6A, D). These results provide evidence that *Uhrf1* plays a critical role in tumor initiation and development in both DEN/CCl_4_-induced and *Myc*-driven HCC models. Therefore, UHRF1 may be a promising therapeutic target for HCC with different molecular pathogenesis.

To date, no specific inhibitor has been developed for UHRF1. Hinokitiol, a tropolone-related natural compound with antitumor activity, has been recently demonstrated to induce DNA demethylation via UHRF1 inhibition in colon cancer cells [33]. Thus, we tested whether hinokitiol could act as a therapeutic agent for HCC. In vitro application of hinokitiol significantly attenuated the proliferation of HCC cell lines in a dose-dependent manner while downregulating the protein levels of UHRF1, GLI1, CD44, and CD133 (Fig. 6E and S7A). Administration of hinokitiol to *Myc* transgenic mice did not cause obvious body weight loss or liver injury (serum markers AST and ALT), despite the slight elevation of alkaline phosphatase (Fig. S7B and C). Hinokitiol treatment substantially retarded *Myc*-driven tumorigenesis and decreased the expression of UHRF1, GLI1, CD44, CD133, and MYC (Fig. 6F–H). Hepatocyte differentiation genes were upregulated following hinokitiol treatment, exhibiting a pattern analogous to UHRF1 depletion (Fig. 6I). While hinokitiol reduces the expression of both UHRF1 and GLI1, it remains unclear whether the tumor-suppressing function of hinokitiol is solely dependent on UHRF1 inhibition. To address this question, it would be necessary to administer it to hepatocyte-specific *Uhrf1* knockout mice to observe if it further suppresses tumor growth. However, due to the limited tumor-forming ability of *Uhrf1*^*HKO*^ mice, this experimental strategy was not viable. Together, these results suggested that hinokitiol inhibited UHRF1 expression and subsequent self-renewal, thereby serving as a potential therapeutic option for HCC.

### UHRF1 and GLI1 are upregulated in HCC tissues and correlated with poor prognosis

**Fig. 7:**
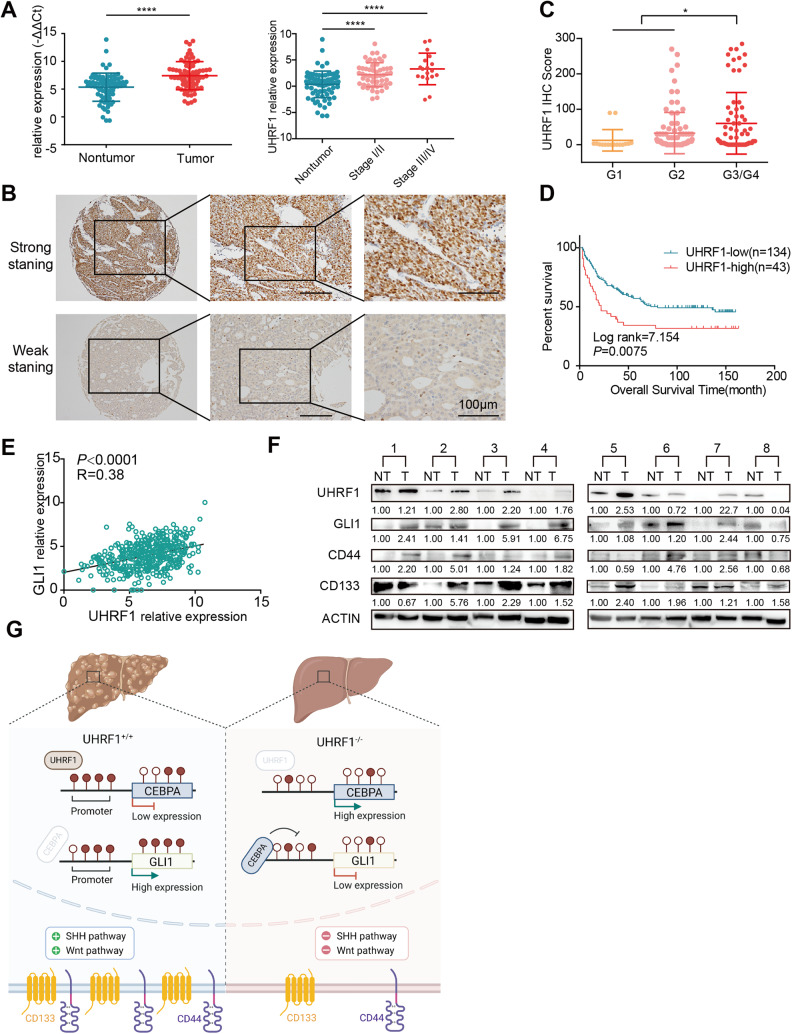
UHRF1 and GLI1 are upregulated in HCC tissues and correlated with poor prognosis. **A** The expression of UHRF1 was analyzed by RT-PCR in our in-house HCC cohort 1 (*n* = 75) (paired Student’s *t* test) (left). UHRF1 mRNA expression in patients with different TNM stages (unpaired Student’s *t* test) (right). **B** Immunohistochemistry staining of UHRF1 in cohort 2 HCC tissue microarray (*n* = 177). Enlarged images are shown in the right panel. **C** IHC scores of UHRF1 protein expression in patients with different neoplasm histological grades in the clinical cohort 2 HCC tissue microarray. **D** Kaplan–Meier overall survival curves of cohort 2 HCC patients with normal or overexpressed UHRF1 (IHC score >50). **E** The mRNA expression of GLI1 positively correlated with UHRF1 (TCGA-LIHC dataset). **F** Detection and quantification of UHRF1, GLI1, CD44, and CD133 expression in HCC tumor (T) tissues and their matched nontumor (NT) tissues by WB analysis. **G** Schematic diagram showing that UHRF1 inhibition epigenetically reprograms CSCs to suppress tumorigenesis and CSC features through GLI1/Hedgehog and Wnt signaling in liver cancer. This figure was created with BioRender software (biorender.com). Mean ± SD. ^∗^*P* < 0.05, ^∗∗∗∗^*P* < 0.0001.

The clinical significance of UHRF1 was further evaluated in our in-house HCC cohort. As detected by qPCR and WB, UHRF1 was significantly increased in tumor samples compared to nontumor samples (Fig. 7A, F). In addition, UHRF1 expression was associated with adverse clinicopathological features and poor prognosis. HCC cases with high histological grades were poorly differentiated and expressed higher levels of UHRF1 (Fig. 7A–D). Similar prognostic and clinicopathological significance of UHRF1 was determined by analyzing the TCGA-LIHC dataset (Fig. S8A, B, and E). In addition, consistent with our experimental results that UHRF1 transcriptionally activated GLI1, a positive correlation between UHRF1 and GLI1 expression was observed in the TCGA HCC dataset (Fig. 7E). Similar to UHRF1, high expression of GLI1 in cancerous tissues was also related to advanced tumor stage, poor histological differentiation, and adverse prognosis (Fig. S8C and D). The protein levels of UHRF1, GLI1, CD133, and CD44 displayed similar expression patterns in HCC tumor and paired nontumor tissues, and overexpression (defined as a twofold increase in tumor) of these proteins was observed in nearly 50% of the samples. (Fig. 7F). These results suggest that the expression of UHRF1 and GLI1 is clinically associated with tumor differentiation, stemness, and prognosis. It may be essential to determine UHRF1 expression in HCC tissues to identify patients who are more likely to benefit from UHRF1-targeting therapy before making a clinical decision. In summary, we demonstrated that UHRF1 expression increased during HCC progression. Moreover, UHRF1 functions as an epigenetic regulator, the depletion of which reprograms liver CSCs toward differentiation and tumor suppression via Hedgehog/GLI1 and Wnt signaling (Fig. 7G).

## Discussion

To date, progress in liver cancer treatment has been limited because of the poor response of HCC cells to current therapies [2]. Strategies that reverse methylation alterations offer unique opportunities for cancer cell reprogramming, which is valuable for the development of new treatments. 5-Azacytidine (5-AZA), a specific inhibitor of DNA methylation, has shown clinical benefits in animal models of hematologic malignancies and solid tumors [34]. These nucleotide analogs are incorporated into replicating DNA, where they irreversibly inactivate DNMT1, DNMT3A, and DNMT3B. However, their notable toxicity to normal cells limits their clinical application. Moreover, constitutive deletion of *Dnmt1* results in early embryonic lethality in mice [35], and liver-specific deletion of *Dnmt1* causes liver injury and structural abnormalities [36]. Further research is required to address the specificity of cancer cell reprogramming, undesirable gene re-expression, and possible side effects on nonneoplastic cells. In contrast to DNMT1, liver-specific *Uhrf1* knockout mice develop normally into viable adults without measurable liver injury or developmental defects [22]. We showed that genetic knockout of UHRF1 markedly attenuated hepatocarcinogenesis in both DEN/CCl_4_-induced and *Myc*-driven HCC mouse models. Targeting UHRF1 achieves higher specificity for HCC cell reprogramming and causes fewer side effects, substantiating its potential to act as a target for HCC therapy.

UHRF1 loss of function induces global hypomethylation, which epigenetically reprograms cancer cells. Similar to other cancers, HCC is characterized by global DNA hypomethylation and regional promoter hypermethylation-mediated TSG silencing [29, 37]. We found that after UHRF1 knockdown, the upregulated genes had a higher percentage of DMRs located in the promoter regions than the downregulated genes. Moreover, hypomethylation induced by UHRF1 knockdown upregulated a higher proportion of TSGs than oncogenes, suggesting that UHRF1 knockdown tends to re-express TSGs. In contrast, gene body methylation has been reported to be positively correlated with expression [38]. Consistent with this, we found that after UHRF1 knockdown, the downregulated genes had a higher degree of demethylation in the gene body region than the upregulated genes. Interestingly, not all gene promoter/body DMRs were negatively or positively correlated with gene expression. The demethylation of these cis-elements was only a necessary but not sufficient condition for them to gain functionality. DNA methylation coordinates with other epigenetic modifications to determine chromatin accessibility. Moreover, the presence of the corresponding trans-elements (such as highly tissue-specific transcription factors) is also a requirement for their action. This may explain why UHRF1 overexpression or deletion in previous studies generated inconsistent effects in different tissues, organisms, and pathophysiological states. Here, we demonstrated that UHRF1 deletion caused global DNA hypomethylation, which activated CEBPA and subsequently repressed GLI1 expression. This finding seems to contradict a previous study, in which Yan et al. found that PGC7 sequestered UHRF1 from the nuclei to the cytoplasm and caused global mCG hypomethylation as well as GLI1 activation [39]. This discrepancy implies that the regulation of DNA methylation is intricate and nuanced in cancer cells. Although both of them induce hypomethylation, the direct loss of UHRF1 function may produce a DNA methylation pattern different from that of PGC7-induced UHRF1 sequestration from the nucleus to the cytoplasm, let alone the difference from 5-AZA treatment and DNMT1 inhibition. Through integrated RNA-seq and whole-genome bisulfite sequencing, the current study depicted the methylome upon UHRF1 depletion at base resolution and revealed transcriptional reprogramming toward differentiation both in vitro and in vivo.

Epigenetic reprogramming triggered by UHRF1 silencing modulates the properties of liver CSCs. Mechanistically, depletion of UHRF1 in HCC cells inhibits GLI1 and Hedgehog signaling. This is accompanied by selective repression of master transcription factors for HCC stem-like cell identity, such as SOX2 and MYC, and upregulation of differentiation effectors. Regulatory loops between GLI1, SOX2, and MYC have been elucidated [40, 41]. We also found elevated UHRF1 and GLI1 expression in *Myc* knock-in mouse livers, and *Uhrf1* knockout, in turn, suppressed the expression of GLI1 and MYC. Blunting UHRF1 activity rebalanced the liver CSC transcriptome toward differentiation and tumor suppression by affecting the transcript levels of master transcription factors.

In summary, our study reveals UHRF1 as an oncogenic driver that confers CSC properties in HCC. Inhibition of UHRF1 through genetic or pharmacological means reduces CSC-like properties and suppresses hepatocarcinogenesis in mouse models. These findings underscore UHRF1 as a promising target for developing treatment strategies for liver cancers.

## Supplementary information

Supplementary Figures and Legends

Supplementary Information - Materials and Methods

Supplemental Material - Original Blots

checklist

## Data Availability

The data generated in this study are publicly available in the Sequence Read Archive (SRA) database at PRJNA895176.
