# Supplementary material for: UHRF1 inhibition epigenetically reprograms cancer stem cells to suppress the tumorigenic phenotype of hepatocellular carcinoma
Source: Cell Death Dis. 2023 Jun 28;14(6):381. doi: 10.1038/s41419-023-05895-w (PMC10307895; doi:10.1038/s41419-023-05895-w)
Supplement: Supplementary file 1 — Supplementary Figures and Legends [file 41419_2023_5895_MOESM1_ESM.pdf]

Supplementary Figures and Legends

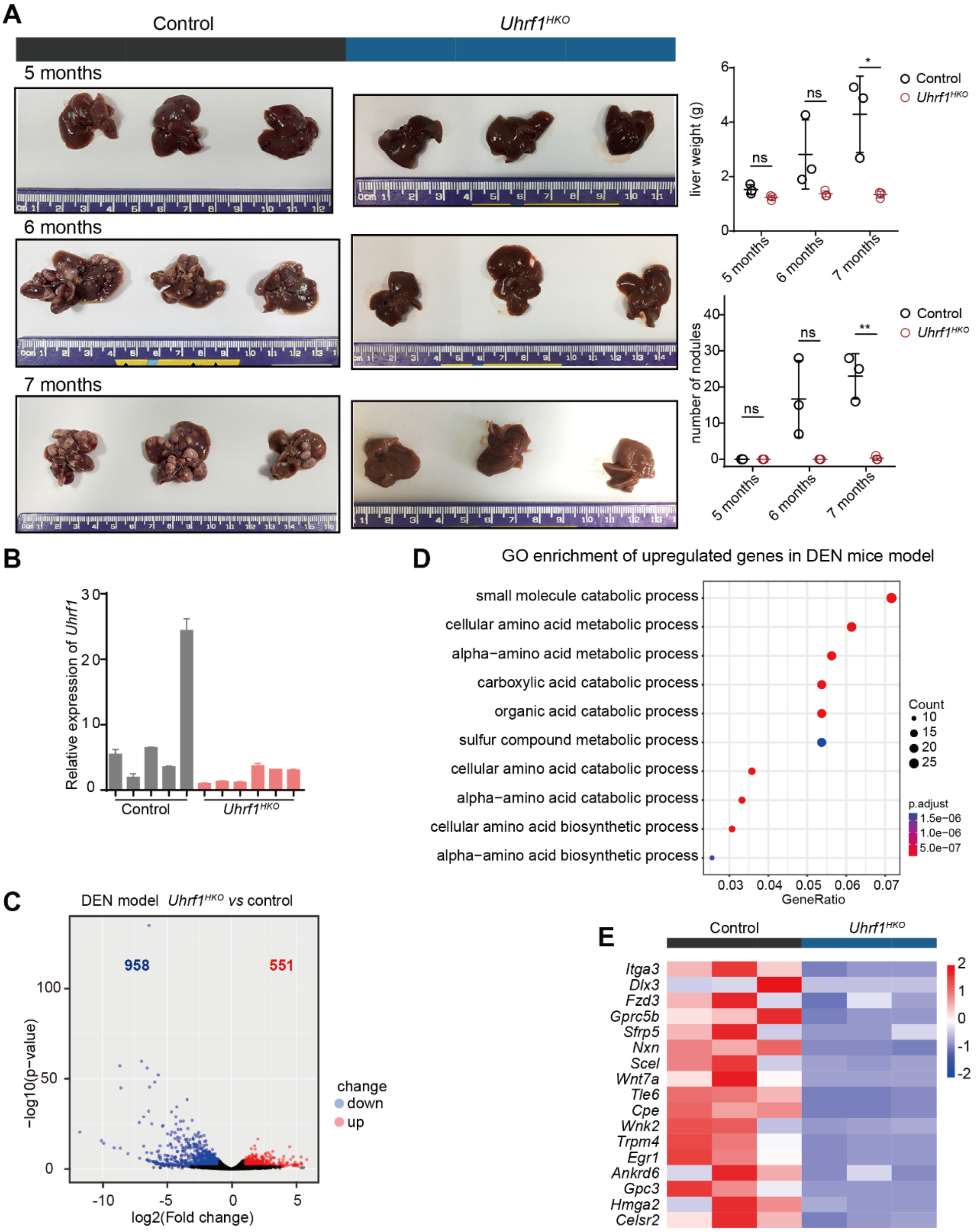

Fig. S1. *Uhrf1^{HKO}* mice exhibit inactivation of Wnt signaling.

(A) Images of mouse liver tissues from the indicated groups at different time points of DEN/CCl_4-induce HCC model.

(B) The mRNA expression of *Uhrf1* in livers of mice as indicated (related to

Fig.1A).

(C) Volcano plot for differentially expressed genes between *Uhrf1*^{HKO} mice compared with control mice.

(D) GO analysis of upregulated genes in *Uhrf1*^{HKO} mice compared with control mice.

(E) Heatmap displays Wnt signaling genes that were downregulated in *Uhrf1*^{HKO}. The color of each cell shows the Z score (log2 of relative abundance scaled by SD) of the mRNA in that sample.

Mean ± SD. *P* values were determined using unpaired Student's t test. $*P<.05$, $**P<.01$. Ns: not significant.

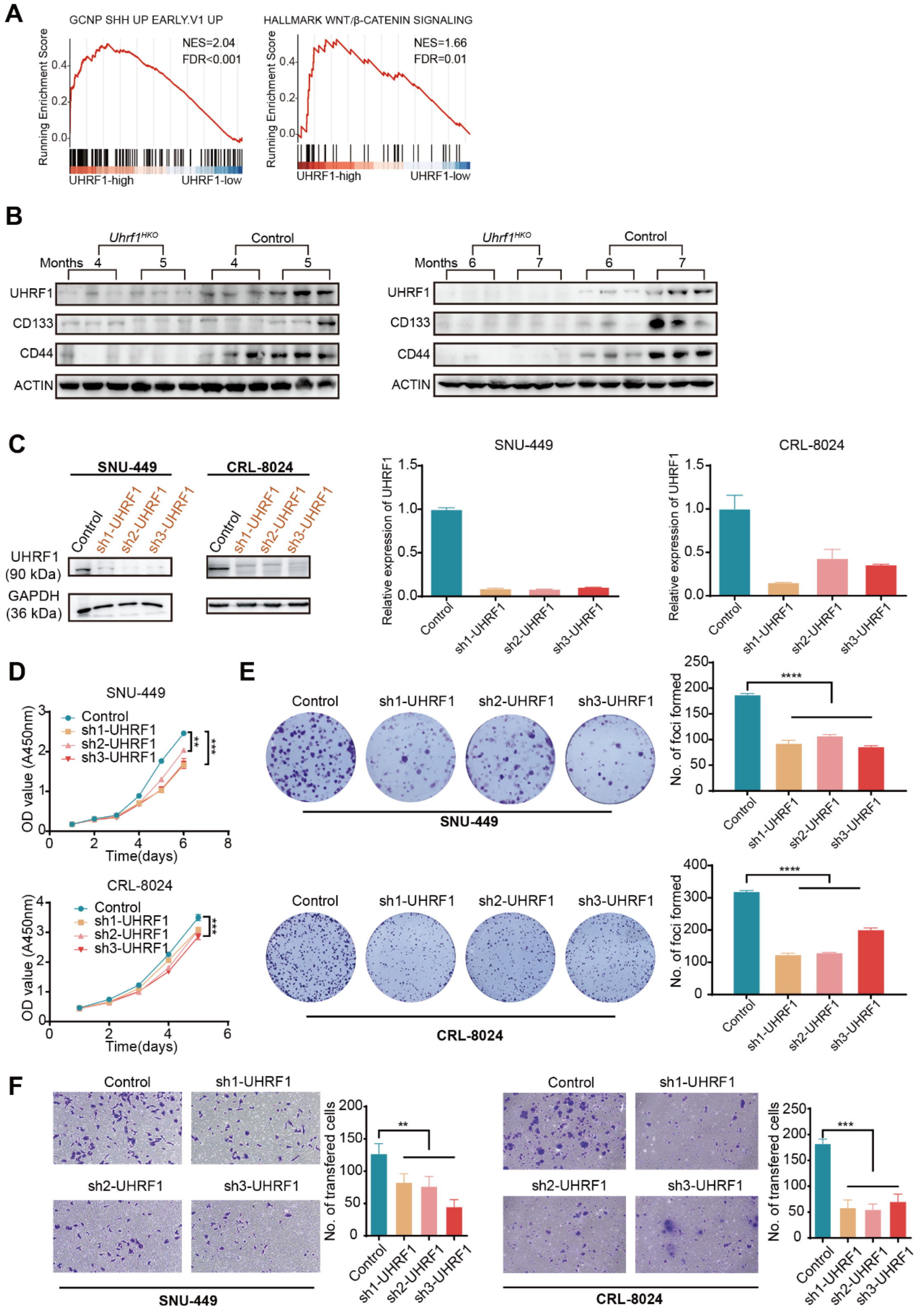

Fig. S2. UHRF1 silencing/KO abolishes the tumorigenic potential of HCC cells.

(A) GSEA analysis revealed that *UHRF1* expression is positively correlated with

Hedgehog and Wnt signaling pathways.

(B) Immunoblotting analysis of the indicated proteins in liver tissues at different time points.

(C) Knock-down of UHRF1 was detected by WB and qPCR. GAPDH was used as loading control.

(D) CCK8 assay assessed viability of the indicated stable cell lines.

(E) Representative images and quantification of foci formation induced by the indicated stable cell lines (n=3).

(F) Representative images and statistical results of transwell assay of the indicated stable cell lines (n=3).

Mean ± SD. *P* values were determined using unpaired Student's t test. $^{**}P < .01$, $^{***}P < .001$, $^{****}P < .0001$.

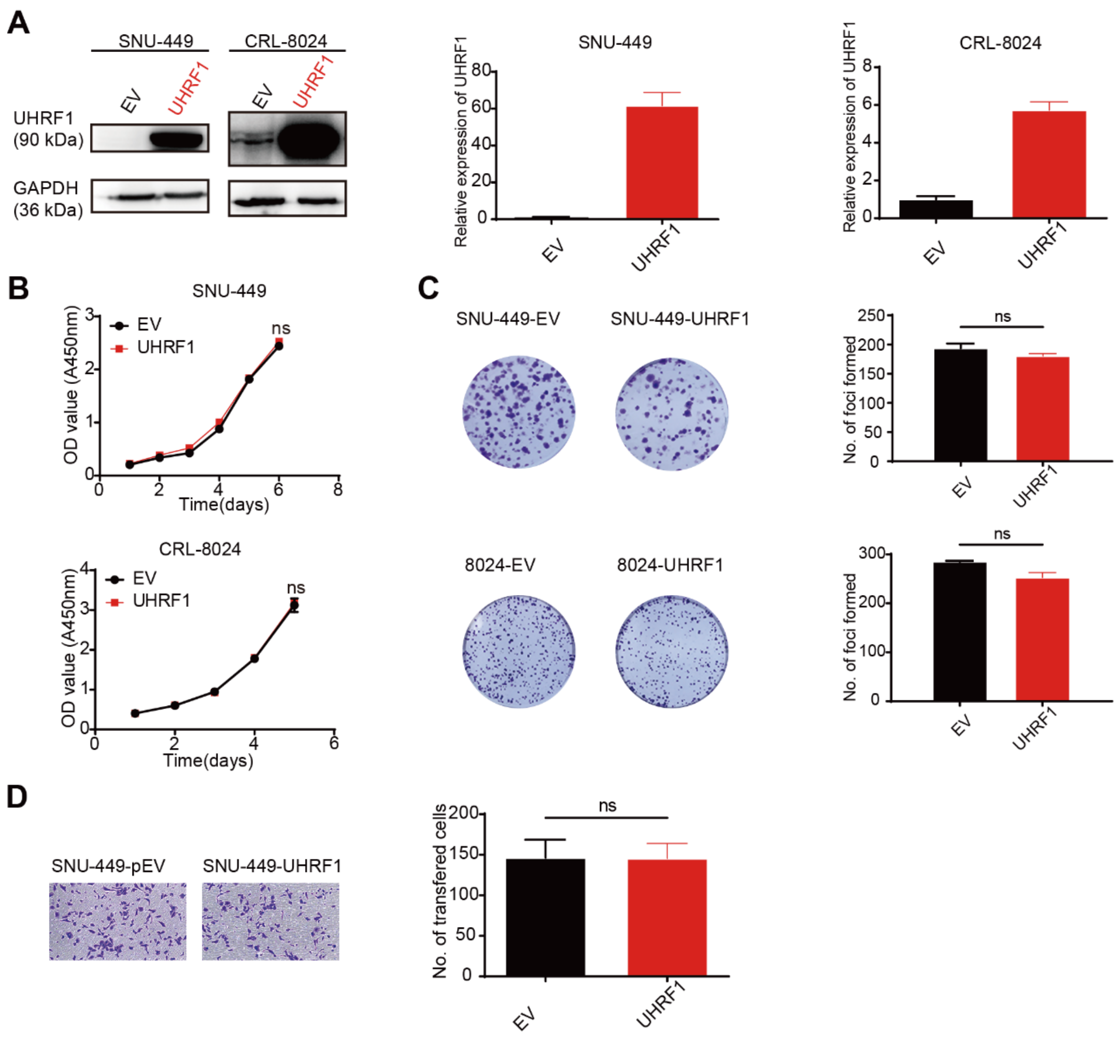

Fig. S3. UHRF1 overexpression shows no significant effect on tumorigenicity of HCC cell lines.

(A) Overexpression of UHRF1 was verified by western blotting and qPCR in SNU-449 and CRL-8024 cells. GAPDH was used as loading control.

(B) CCK8 assay assessed viability of the indicated cells.

(C) Representative images and quantification of foci formation induced by the indicated cells (n=3).

(D) Representative images and statistical results of transwell assay of the indicated cells (n=3).

Mean ± SD. *P* values by unpaired Student's t test. Ns: not significant.

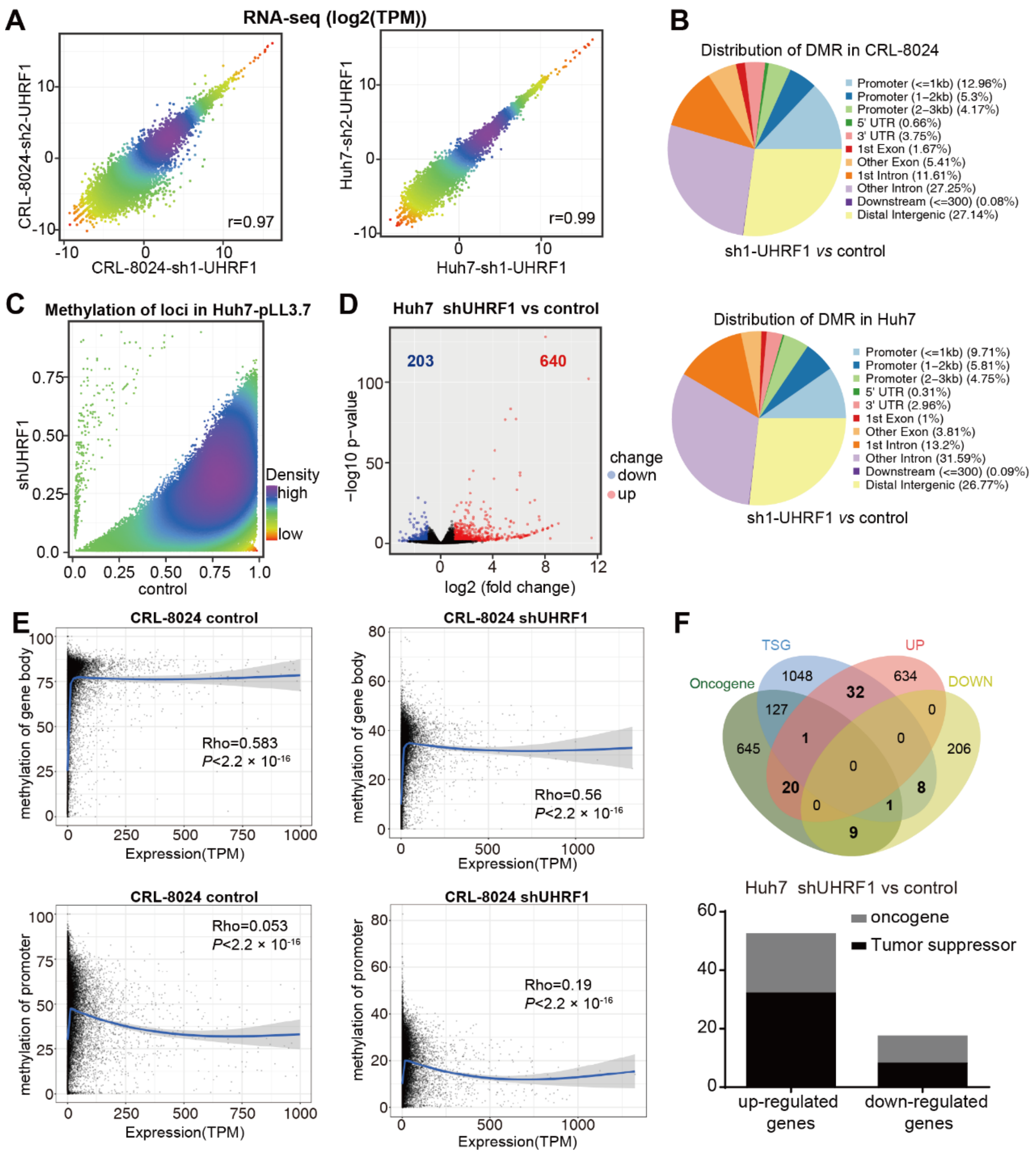

Fig. S4. UHRF1 knockdown induces drastic changes in transcriptome and DNA methylome.

(A) Scatter plots of gene expression values (log2 (TPM)) in the indicated samples.

(B) Genomic distribution of DMR in HCC cell lines.

(C) Density scatter plot of DNA sites with different DNA methylation levels between control and UHRF1 knockdown Huh7 cells.

(D) Volcano plot for differentially expressed genes between control and UHRF1 knockdown Huh7 cells.

(E) Scatter plot and trend line showing the correlation between DNA methylation and gene expression (TPM) in control and UHRF1 knockdown CRL-8024 cells. The y-axis represents the numeral methylation in gene body or promoter regions, and the x-axis represents the gene expression (TPM).

(F) Venn diagram among four data sets in Huh7 cells. The overlapping genes are described in bar chart.

P values by Spearman's correlation coefficient.

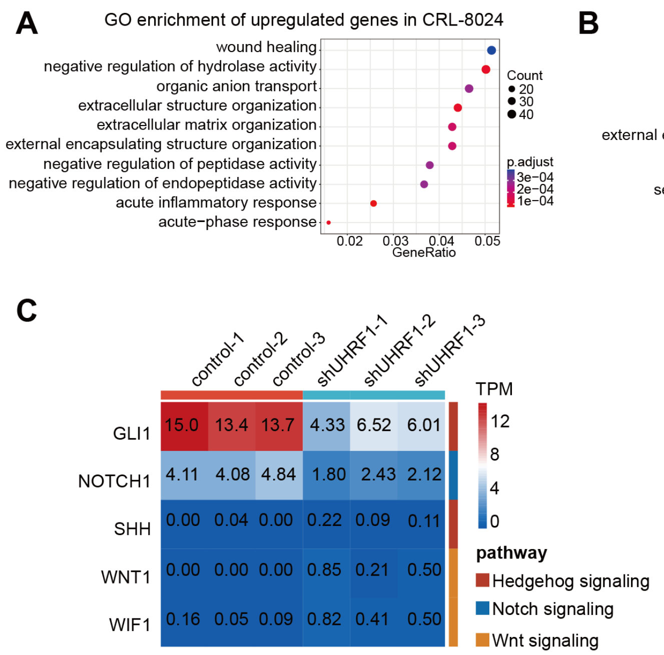

Fig. S5. UHRF1 silencing leads to transcriptional changes in stemness and differentiation related genes.

(A) GO analysis of upregulated genes in CRL-8024 UHRF1 knockdown group compared with CRL-8024 control group.

(B) GO analysis of upregulated genes in Huh7 UHRF1 knockdown group compared with Huh7 control group.

(C) Heatmap displays relative expression of overlapped genes related to Fig. 4C.

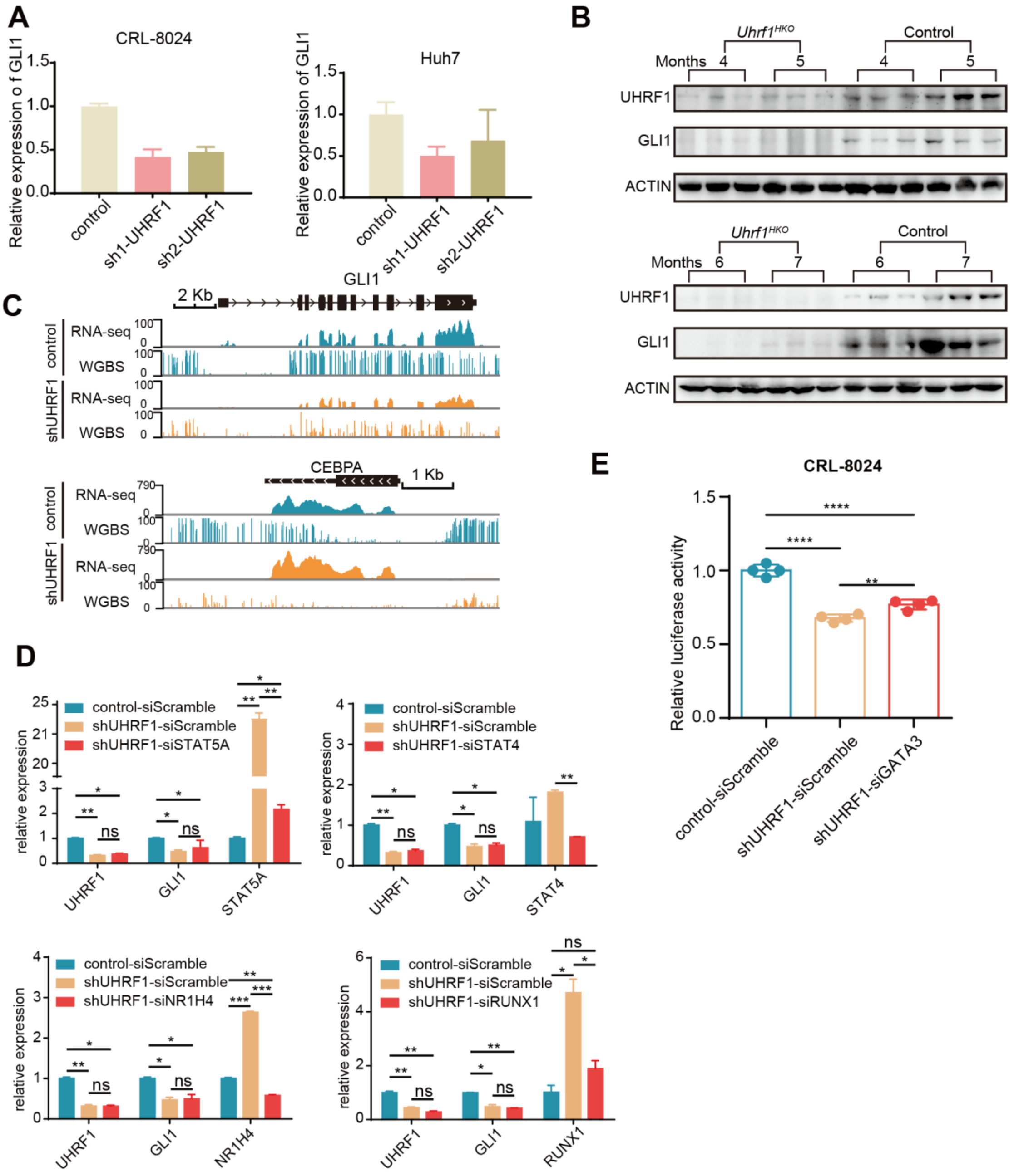

Fig. S6. UHRF1 silencing down-regulated GLI1 through candidate transcription factors.

(A) The mRNA expression of GLI1 in cell lines as indicated.

(B) Protein level of GLI1 in liver tissues from *Uhrf1^{HKO}* mice and corresponding control mice at different time points of DEN/CCl_4 induced HCC model.

(C) RNA-seq signals and methylated CpG levels at the *GLI1*/*CEBPA* locus of

UHRF1 knockdown and control CRL-8024 cells.

(D) The mRNA expression of the indicated genes in CRL-8024 cells after STAT5, STAT4, NR1H4 or RUNX1 silencing.

(E) *GLI1* promoter was cloned into the pGL3-basic vector and co-transfected with siGATA3 or scramble siRNA for dual-luciferase assays (n=4).

Mean ± SD. *P* values by unpaired Student's t test. ∗*P* <.05, ∗∗*P* <.01, ∗∗∗*P* <.001, ∗∗∗∗*P* <.0001. Ns: not significant.

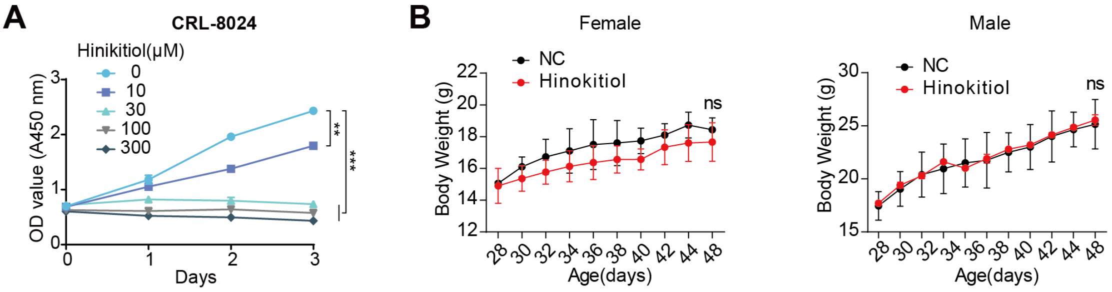

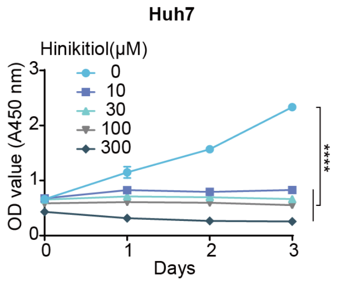

C

Test	Results	Normal Range
ALT	33.3±10.3	8-90 U/L
AST	120.2±8.4	75-145 U/L
Alkaline Phosphatase	186.4±37.0 ↑	70-110 U/L
Total Protein	60.2±19.68	45-70 g/L
Albumin	25.6±0.8	24-45 g/L

Fig. S7. *In vitro* and *in vivo* effects of hinokitiol treatment.

(A) Cell viability was detected by CCK8 assay in cells treated with gradient concentration of hinokitiol as indicated.

(B) Statistics of body weight of mice treated with hinokitiol. n=4-6. Ns.no significant.

(C) Liver function test using serum collected from mice after 5 weeks of treatment with hinokitiol (25 mg/kg, n=3).

Mean ± SD. *P* values by unpaired Student's t test. $**P<.01$, $***P<.001$, $****P<.0001$. Ns: not significant.

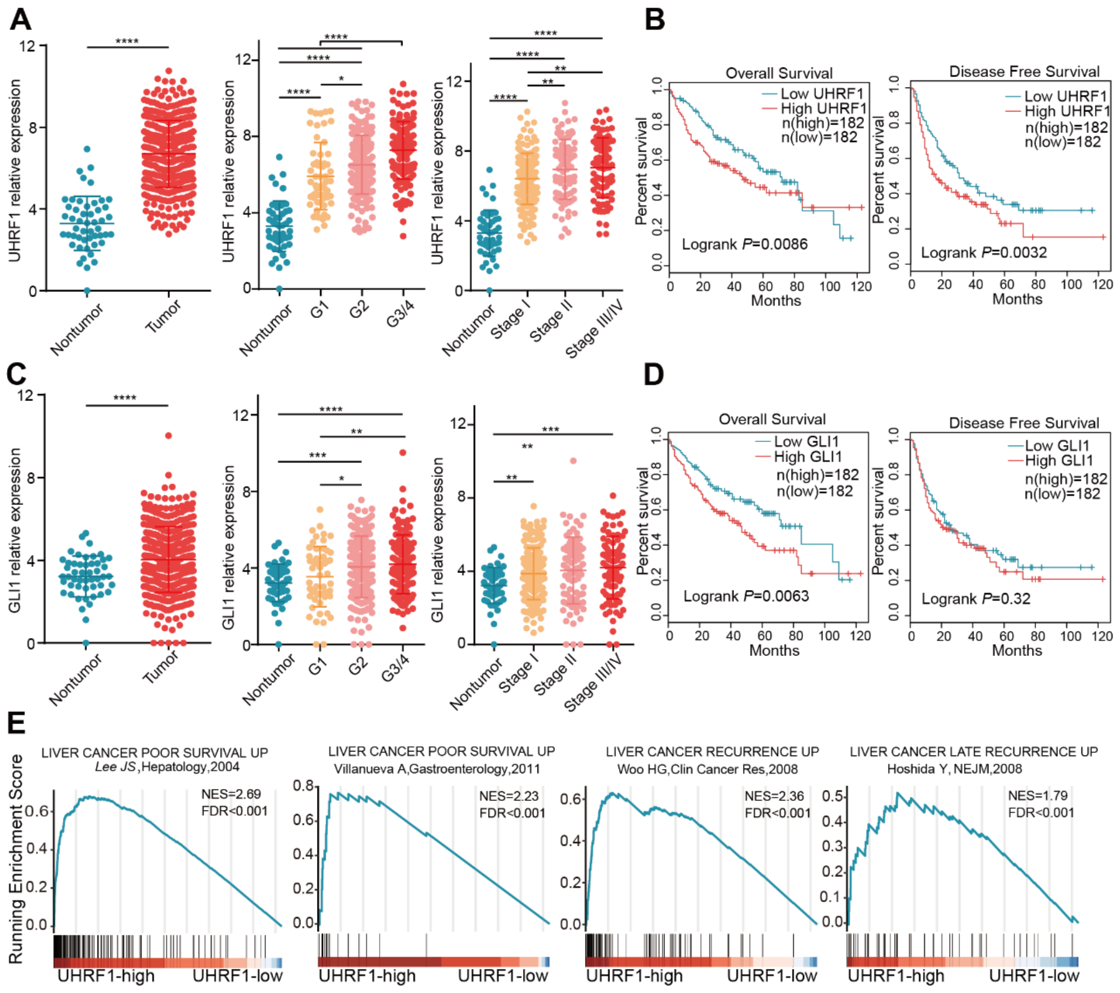

Fig. S8. Overexpression of *UHRF1* and *GLI1* associates with poor prognosis in TCGA-LIHC dataset.

(A) UHRF1 mRNA levels in nontumor samples and HCC samples with different neoplasm histologic grades and tumor stages (TCGA-LIHC).

(B) Kaplan-Meier overall survival curves and disease-free survival curves of HCC patients with high UHRF1 expression (n= 182) or low UHRF1 expression (n= 182) (TCGA-LIHC dataset).

(C) GLI1 mRNA levels in non-tumor samples and HCC samples with different neoplasm histologic grades and tumor stages (TCGA-LIHC).

(D) Kaplan-Meier overall survival curves and disease-free survival curves of

HCC patients with high GLI1 expression (n= 182) or low GLI1 expression (n= 182) (TCGA-LIHC dataset).

(E) Gene Set Enrichment Analysis (GSEA) revealed UHRF1 is associated with poor outcome and recurrence in HCC (TCGA-LIHC dataset).

Mean ± SD. *P* values by unpaired Student's t test. $*P < .05$, $**P < .01$, $***P < .001$, $****P < .0001$.
