# Supplementary material for: UHRF1 inhibition epigenetically reprograms cancer stem cells to suppress the tumorigenic phenotype of hepatocellular carcinoma
Source: Cell Death Dis. 2023 Jun 28;14(6):381. doi: 10.1038/s41419-023-05895-w (PMC10307895; doi:10.1038/s41419-023-05895-w)
Supplement: Supplementary file 2 — Supplementary Information - Materials and Methods [file 41419_2023_5895_MOESM2_ESM.docx]

**Supplementary Information**

**Materials and Methods**

***Cell lines***

CRL-8024 was obtained from Institute of Virology, Chinese Academy of Medical Sciences (Beijing, China). 293T was obtained from National Collection of Authenticated Cell Cultures (Shanghai, China). HCC cell line, Huh7, was provided by JCRB Cell Bank (Ibaraki, Japan). SNU-449 was obtained from the American Type Culture Collection (ATCC, USA). All cell lines were authenticated by short tandem repeat profiling and routinely tested for the absence of mycoplasma contamination. Cells were cultured in Dulbecco’s modified Eagle’s medium (DMEM) or RPMI-1640 (Gibco, USA), supplemented with 10% fetal bovine serum (Gibco), and 1% penicillin/streptomycin mixture (Gibco). All cell lines used in this study were incubated at 37°C in a humidified incubator containing 5% CO_2_.

***Plasmids, Lentivirus Production and Cell Infection***

We constructed UHRF1 or GLI1 overexpression vector with pLenti6 plasmid (Invitrogen, USA). UHRF1-shRNA expression vectors and the scrambled shRNA control were constructed with pLL3.7 plasmid (Addgene, USA). These plasmids together with lentivirus packaging vectors from pLenti6/V5 Directional TOPO Expression Kit (Invitrogen) were co-transfected into 293T cells. Two days later virus-containing supernatants were then collected for subsequent transduction into cell lines. Stable overexpression or knockdown cells were selected with blasticidin (Sigma-Aldrich, Germany) and puromycin (ThermoFisher Scientific, USA), respectively. For dual-luciferase reporter assay, GLI1 promoter (-2000/+100bp) was cloned into pGL3 plasmid (Promega, USA), the plasmid was transferred into pll3.7 CRL-8024 or shUHRF1 CRL-8024 cell lines 24h after siCEBPA or siScramble treatment, 24h later cells were collected for fluorescence detection. Primers and UHRF1-shRNAs sequences are listed in Supplementary Table 3.

***Liver function test***

Venous blood samples were collected and centrifuged at 860 ×g for 15 min to harvest serum. Serum samples were used to measure albumin, total protein, alkaline phosphatase, aspartate aminotransferase (AST) and alanine aminotransferase (ALT) levels using fully automatic biochemical analyzer (Agilent 2100) according to the manufacturer’s instructions.

***Cell viability assay***

Cells were plated in 96 well plate at 10^3^ cells/well with 100 μL medium, 10 μL CCK8 reagent (MCE, China) was added to the culture medium 4 hours before analysis, OD450 was detected for cell relative viability.

***Foci formation***

Cells were plated in 6 well plate at 10^3^ cells/well with 2mL medium. 7-12 days later cells were washed with PBS three times, fixed with 75% ethanol for 30 mins and dyed purple by gentian violet. The number of foci were calculated by software ImageJ, foci particles between 50 and 1000 were analyzed.

***Transwell migration assay***

Migration assays were performed with Chambers (Corning, USA) according to the manufacturer’s instructions (Corning). 2*10^4^ cells were seeded into transwell inserts with 200 μL medium, cells migrated to the other side of the membrane were dyed purple by gentian violet and counted after 48 hours. The number of migrated/invaded cells was counted in 10 fields under a 20× objective lens.

***Sphere formation assay***

Cells were plated as single cell into 96 well plate at 100 cells/well with 200 μL DMEM/F12 (Hyclone, USA) contains 1% B27 supplement (Thermofisher, USA), 1% N2 supplement (Thermofisher), 20 ng/mL EGF (Thermofisher, USA) and 10 ng/mL bFGF (Thermofisher). After 7 days of growth, spheres were counted. Sphere formation rate = sphere formed cells/seeded cells.

***RNA extraction, reverse transcription and*** ***quantitative real-time PCR***

Cells or tissues were lysed by TRIZOL reagent (Merck, Sigma-Aldrich, Germany) (tissues were pre-ground) according to the manufacturer’s instructions. Total RNA was used to synthesize the first strand of cDNA using Transcriptor High Fidelity cDNA Synthesis (Roche, Switzerland). For PCR analysis, Green Taq Mix (TAKARA, China) were used according to the manufacturers’ instruction. The PCR product was analyzed by electrophoresis in 1% agarose gel. For qRT-PCR analysis, SYBR® Green Premix Kit (TAKARA, China) was used according to the manufacturers’ instruction. Relative changes in gene expression were calculated using the 2 ^-ΔΔCt^ method. Primers sequences are listed in Supplementary Table 3.

***RNA-seq sample preparation and analysis***

UHRF1 knockdown and control CRL-8024 cells (3 replicates) were suspended in TRIzol reagent (Invitrogen) and sent to BGI (Shenzhen, China) for RNA sequencing. RNA-seq libraries prepared using oligo (dT) beads and rRNA removal methods were pooled and sequenced using an BGISEQ platform. Paired-end reads were mapped to the Homo sapiens GRCh38 (hg38) reference genome and using the STAR RNA-seq aligner. Raw reads counts were obtained by featureCounts. Next, we used DESeq2 R package [^1^](#_ENREF_1) for differential gene analysis (fold change > 2 and *p* <.05). GO enrichment analysis was performed using ClusterProfiler R package [^2^](#_ENREF_2). The TPM value was used to assess the expression levels of genes for heatmap plotting.

***Western blot***

Cells or tissues(pre-ground) were lysed for 1h by RIPA liquid and centrifuged for 15 min at 12000 rpm to obtain protein, BCA kit was applied for the measurement of protein concentration, then the lysis was denatured by mixing with loading buffer (SDS and β-mercaptoethanol) and boiling at 95 °C for 5 min. The protein samples were then run in SDS-PAGE 120 V for 2 hours, transferred to the PVDF membranes, blocked by PBST contains 5% milk powder for 2 hours at room temperature. Subsequently, the membranes were incubated with rabbit monoclonal anti-UHRF1 (1:1000 dilution), rabbit monoclonal anti-CD44 (1:2000 dilution), rabbit monoclonal anti-CD133 (1:1000 dilution), rabbit anti-GLI1 (1:1000 dilution), monoclonal anti-ACTIN (1:2000 dilution) overnight at 4 °C. The membranes were sequentially incubated with a secondary antibody for 1 hour at room temperature and ECL kit (Hydrogen peroxide + luminol), then chemiluminescence images were acquired by chemiluminescence imaging analysis system.

***Immunohistochemistry staining***

The TMA slides of liver cancer patients or mice liver paraffin sections were dried overnight at 37 °C, deparaffinized and rehydrated through graded alcohol, immersed in 3% hydrogen peroxide for 20 minutes to block endogenous peroxidase activity, and antigen retrieved by pressure cooking for 3 minutes in EDTA buffer (pH 8.0). The slides then were preincubated with 10% normal goat serum at room temperature for 30 minutes to reduce nonspeciﬁc reaction. Subsequently, the slides were incubated with mouse monoclonal anti-UHRF1 (1:50 dilution) for 12 hours at 37 °C (or 2 hours at room temperature). The slides were sequentially incubated with a secondary antibody for 1 hour at room temperature and stained with DAB (3, 3-diaminobenzidine). Finally, the sections were counterstained with Mayer hematoxylin, dehydrated, and mounted. A negative control was obtained by replacing the primary antibody with a normal murine immunoglobulin G.

Stained IHC specimens are scored with a microscope by pathologists with the H-score, a semi-quantitative score system which calculates a score from 0 to 300 based on both the intensity of tumor cytoplasmic staining and the percentage of cells stained. Intensity is considered "0" for absent expression, "1+" for weak staining, "2+" for moderate staining, and "3+ for strong staining. The H-score is calculated as follows: H-Score = (0 x percentage of cells with absent cytoplasmic staining) + (1 x percentage of "1+" cells) + (2 x precentage of "2+" cells) + (3 x percentage of "3+ cells)

***Immunofluorescence staining***

The mice liver tissue slides or cell slides were fixed with 4% paraformaldehyde, then were preincubated with 10% normal goat serum at room temperature for 30 minutes to reduce nonspeciﬁc reaction. Subsequently, the slides were incubated with mouse monoclonal anti-UHRF1 (1:50 dilution), rabbit monoclonal anti-CD44 (1:200 dilution), rabbit monoclonal anti-CD133 (1:200 dilution), and rabbit anti-GLI1 (1:200 dilution) for 12 hours at 4 °C or 2 hours at 37 °C. The slides were sequentially incubated with a secondary antibody (fluor 555 or fluor 488 labeled) for 1 hour at room temperature and stained with DAPI for 5 min. Then observed and taken photos under confocal microscope, 5 fields of view were taken for each sample, and the proportion of CD44^+^, GLI1^+^, or CD133^+^ cells were calculated.

***Fluorescence-activated cell sorting (FACS)***

Cells were digested with trypsin for 4 min and centrifuged 4 min at 1000*g to collected precipitate, then cells were incubated with anti-CD44 (APC labeled, 1:200 dilution), anti-CD133 (PE labeled, 1:200 dilution) for 15 min at 4°C in dark, sequentially rinsed twice by PBS. Then CD44^high^ & CD133^high^ cells were sorted by flow cytometry.

***Dual-luciferase assay***

Glowworm luciferase plasmid and Renilla luciferase plasmid were co-transfected into shUHRF1 or control CRL-8024 cells at a ratio of 10:1. Cells were pretreated with control siRNA or siCEBPA/siGATA3. 24h later cells were lysed with lysis buffer. Dual-Luciferase Reporter Assay kit (Promega, USA) was used to detect relative luciferase activity.

***Motif enrichment analysis and transcription factor analysis***

Package Homer was used for motif enrichment analysis of interested sequences, and transcription factor corresponding to each discovered motif were obtained. Predict transcription factor of interested genes were obtained online using AnimalTFDB3.0 (<http://bioinfo.life.hust.edu.cn/HumanTFDB/#!/>) [^3^](#_ENREF_3).

***Online databases***

TSG genes and oncogenes were obtained from TSGene and ONGene respectively [^4^](#_ENREF_4) [^5^](#_ENREF_5).

***Whole-genome bisulfite sequencing (WGBS) sample preparation and* *analysis***

DNA extraction and purification from control and shUHRF1 CRL-8024 cells. Extracted genomic DNA was prepared for bisulfite sequencing, the DNA fragments were adapted to barcoded methylated adaptors (Illumina), the adapted DNA was converted using the EZ DNA Methylation Lightning kit (Zymo Research) and then amplified for ten cycles using iQ SYBR Green Supermix (Bio-Rad). Libraries were pooled and size-selected using 6% TBE polyacrylamide gels, then were sequenced using the Illumina HiSeq platform for paired ends for 100–111 cycles and the Illumina MiSeq platform for paired ends for 75 cycles. The alignment of sequencing data was performed by Bismark [^6^](#_ENREF_6). R package DSS [^7^](#_ENREF_7) was used to seek differentially methylated locus (*p*<0.01) and differentially methylated regions (Δ>0.1, *p*<.01). Strict Wohlde test based on beta negative binomial distribution was used to examine differences.

***TCGA data analyses***

The HCC transcriptome data were obtained from The Cancer Genome Atlas Liver Hepatocellular Carcinoma (TCGA_LIHC) project. Gene Set Enrichment Analysis (GSEA) (<http://www.broadinstitute.org/gsea>) was performed to identify associated molecular pathways.

***Statistics***

SPSS 22.0 or GraphPad Prism 8.0 was used for statistical analyses. *P* < 0.05 was considered statistically significant. An unpaired Student's t-test was used to examine differences between two groups. Cox–Mantel log-rank test was used for Kaplan–Meier survival curves. Heatmap, scatter plot, and volcano diagram were generated using the R package ([www.r-project.org](http://www.r-project.org)).

**Supplemental Tables**

**Supplementary Table 1. Plasmid information**

| **Plasmid name** | **Catalog number** |
| --- | --- |
| pLenti6 | Addgene plasmid #128062 |
| pLL3.7 | Addgene plasmid #11795 |
| *pRL-TK | Promega plasmid #E2241 |
| *pGL3-Basic | Promega plasmid #E1751 |

* The plasmids used for the study were obtained from Professor Xijun Ou.

**Supplementary Table 2. Antibodies**

| **Antibody** | **Dilution** | **Manufacturer** | **Catalog number** |
| --- | --- | --- | --- |
| Mouse monoclonal anti-UHRF1 | 1:50 IHC  1:100 IF | Santa Cruz | Cat# sc-373750 |
| Mouse monoclonal anti-UHRF1 | 1:1000 WB | Abcam | Cat# 12387 |
| Rabbit polyclonal anti-CD133 | 1:1000 WB  1:200 IF | Proteintech | Cat# 18470-1-AP |
| PE anti-CD133 monoclonal antibody | 1:100 Flow | Miltenyi Biotec | Cat# 130-113-670 |
| Rabbit polyclonal anti-CD44 | 1:1000 WB  1:200 IF | Proteintech | Cat# 15675-1-AP |
| APC anti-CD44 monoclonal antibody | 1:100 Flow | Miltenyi Biotec | Cat# 130-113-893 |
| Rabbit polyclonal anti-GLI1 | 1:1000 WB | Novusbio | Cat# NB600-600 |
| Rabbit monoclonal anti-c-Myc | 1:2000 WB | Abcam | Cat# 18583 |
| Mouse monoclonal anti-GAPDH | 1:5000 WB | Cell Signaling Technology | Cat# 51332 |
| Mouse monoclonal anti-ACTIN | 1:5000 WB | Cell Signaling Technology | Cat# 3700 |
| Alexa 488 anti-Mouse Secondary Antibody | 1:500 IF | Thermo Fisher Scientific | Cat# A-11029 |
| Alexa 555 anti-Rabbit Secondary Antibody | 1:500 IF | Thermo Fisher Scientific | Cat# A-21428 |

**Supplementary Table 3. Sequences of nucleotides**

| **Sequences name** | **Sequences** |
| --- | --- |
| qPCR Primer |  |
| UHRF1  (Human) | forward primer GATGATGTGGACCATGGGA  reverse primer GATCACAAGACTGTTCCGC |
| UHRF1  (Mouse) | forward primer CGGAGGATGACATCATGTACCA  reverse primer TCCCATGGTATCACAGTGCG |
| GLI1  (Human) | forward primer AACTCCACAGGCATACAGG  reverse primer TACACAGATTCAGGCTCACG |
| GLI1  (Mouse) | forward primer GCTTTCATCAACTCTCGCT  reverse primer TCCTAAAGAAGGGCTCATGG |
| c-MYC  (Mouse) | forward primer CTGTACCTCGTCCGATTCC  reverse primer GCTCTTCTTCAGAGTCGCT |
| RUNX1  (Human) | forward primer CATCGCTTTCAAGGTGGTG  reverse primer ATTTCTCAGCTCAGCCGAG |
| CD44  (Human) | forward primer CTTCAATGCTTCAGCTCCA  reverse primer ATGGTAATTGGTCCATCAAAGG |
| CD44  (Mouse) | forward primer GTACCTTACCCACCATGGAC  reverse primer TTCCTTCTATGAACCCATACCTG |
| CD133  (Human) | forward primer AGGAGGCGGAATTCTTGAC  reverse primer TTGGTCTCCTTGATCGCTG |
| CEBPA  (Human) | forward primer CTGACCAGTGACAATGACC  reverse primer CCTTGACCAAGGAGCTCTC |
| GATA3  (Human) | forward primer GCTTCACAATATTAACAGACCC  reverse primer TAAACGAGCTGTTCTTGGG |
| STAT4  (Human) | forward primer AAATCAAGACTGGGAGGCA  reverse primer CGACCTAACTGTTCATCCAG |
| STAT5A  (Human) | forward primer TTCAACAGGGAGAACTTGC  reverse primer GTGCTTCTTCAACACCTCC |
| RUNX1  (Human) | forward primer CATCGCTTTCAAGGTGGTG  reverse primer ATTTCTCAGCTCAGCCGAG |
| NR1H4  (Human) | forward primer AAAGCTACCAGGATTTCAGAC  reverse primer AGGAACATAGCTTCAACCG |
| GAPDH  (Human) | forward primer CCACATCGCTCAGACACCAT  reverse primer AGGAACATAGCTTCAACCG |
| NR1H4  (Human) | forward primer AAAGCTACCAGGATTTCAGAC  reverse primer AGGAACATAGCTTCAACCG |
| siRNA |  |
| UHRF1 (Human) | GCCUUUGAUUCGUUCCUUCUU |
| UHRF1 (Human) | GCCUUUGAUUCGUUCCUUCUU |
| CEBPA (Human) | GCAAAUCGUGCCUUGUCAUUU |
| GATA3 (Human) | CAUCCAGACCAGAAACCGAAA |
| STAT4 (Human) | CGCACCAAGAAAGGAAGCAAA |
| STAT5A (Human) | GGACCUUCUUGUUGCGCUUUA |
| RUNX1 (Human) | CCUCGAAGACAUCGGCAGAAA |
| RUNX1 (Human) | GAACCACUCCACUGCCUUUAA |
| NR1H4 (Human) | UGUUGGCUGAAUGCUUGUUAA |
| shRNA |  |
| UHRF1 (Human) | GCCTTTGATTCGTTCCTTCTT |
| UHRF1 (Human) | GCGCTGGCTCTCAACTGCTTT |
| UHRF1 (Human) | ATGTGGGATGAGACGGAATTG |

**Supplementary Table 4. List of** **reagents**

| **Reagents** | **Supplier** | **Cat#** |
| --- | --- | --- |
| Diethylnitrosamine (DEN) | Sigma-Aldrich | N0258 |
| Hinikitiol | MCE | HY-B2230 |
| CCl_4_ | Sigma-Aldrich | 56-23-5 |
| Dual-Luciferase Assay kit | Promega | E1910 |
| SlowFade Antifade Reagents | Thermo Fisher Scientific | S36937 |
| Polybrene | Sigma-Aldrich | H9268 |
| Puromycin | Gibco | A1113803 |
| Blasticidin | Gibco | A1113903 |
| Fetal bovine serum | Gibco | A3160802 |
| Protease Inhibitor Cocktail | Roche | 4693159001 |
| Opti-MEM | Gibco | 31985070 |
| RPMI-1640 | Gibco | C11875500CP |
| DMEM | Gibco | C11995500BT |
| RIPA Buffer | Sigma-Aldrich | R0278 |
| Lipofectamine 3000 | Thermo Fisher Scientific | L3000-015 |
| DAPI | Sigma-Aldrich | D9542 |

**Supplementary references**

1. Love MI, Huber W, Anders S. Moderated estimation of fold change and dispersion for RNA-seq data with DESeq2. *Genome Biol* 2014, **15**(12)**:** 550.

2. Yu G, Wang L-G, Han Y, He Q-Y. clusterProfiler: an R package for comparing biological themes among gene clusters. *Omics: a journal of integrative biology* 2012, **16**(5)**:** 284-287.

3. Hu H, Miao YR, Jia LH, Yu QY, Zhang Q, Guo AY. AnimalTFDB 3.0: a comprehensive resource for annotation and prediction of animal transcription factors. *Nucleic Acids Res* 2019, **47**(D1)**:** D33-d38.

4. Liu Y, Sun J, Zhao M. ONGene: A literature-based database for human oncogenes. *J Genet Genomics* 2017, **44**(2)**:** 119-121.

5. Zhao M, Sun J, Zhao Z. TSGene: a web resource for tumor suppressor genes. *Nucleic Acids Res* 2013, **41**(Database issue)**:** D970-976.

6. Krueger F, Andrews SR. Bismark: a flexible aligner and methylation caller for Bisulfite-Seq applications. *Bioinformatics* 2011, **27**(11)**:** 1571-1572.

7. Feng H, Conneely KN, Wu H. A Bayesian hierarchical model to detect differentially methylated loci from single nucleotide resolution sequencing data. *Nucleic Acids Res* 2014, **42**(8)**:** e69.
