# Supplementary material for: UHRF1 inhibition epigenetically reprograms cancer stem cells to suppress the tumorigenic phenotype of hepatocellular carcinoma
Source: Cell Death Dis. 2023 Jun 28;14(6):381. doi: 10.1038/s41419-023-05895-w (PMC10307895; doi:10.1038/s41419-023-05895-w)

Supplemental Material – Original Blots

Relevant areas for cropped blots in the main and Extended Data figures are shown with a dashed box.

Fig 1B (original blots)

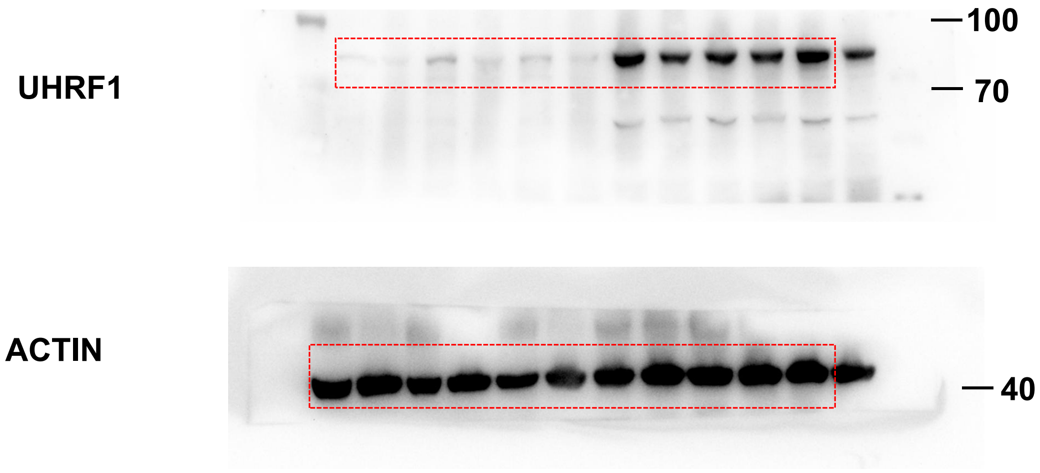

Fig 2D (original blots)

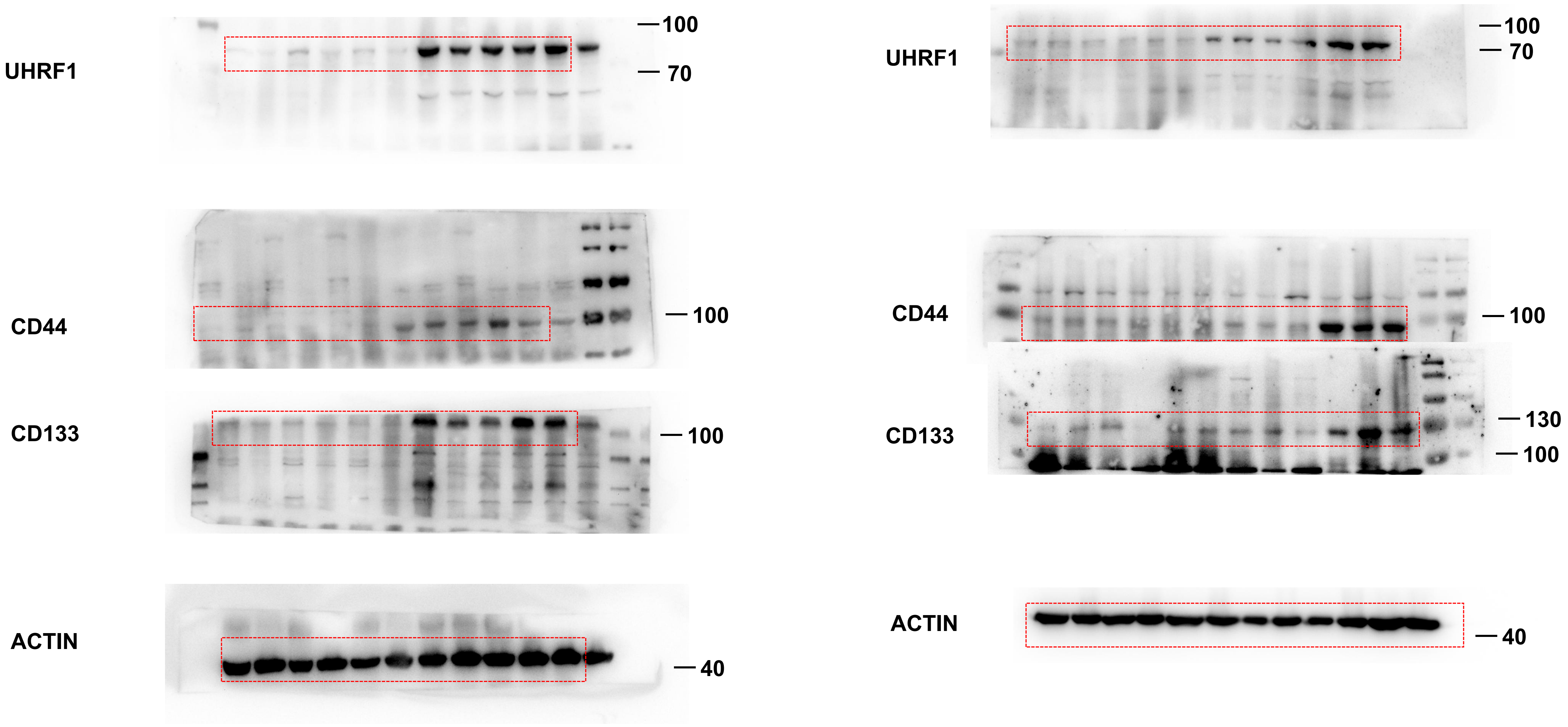

Fig S2B (original blots)

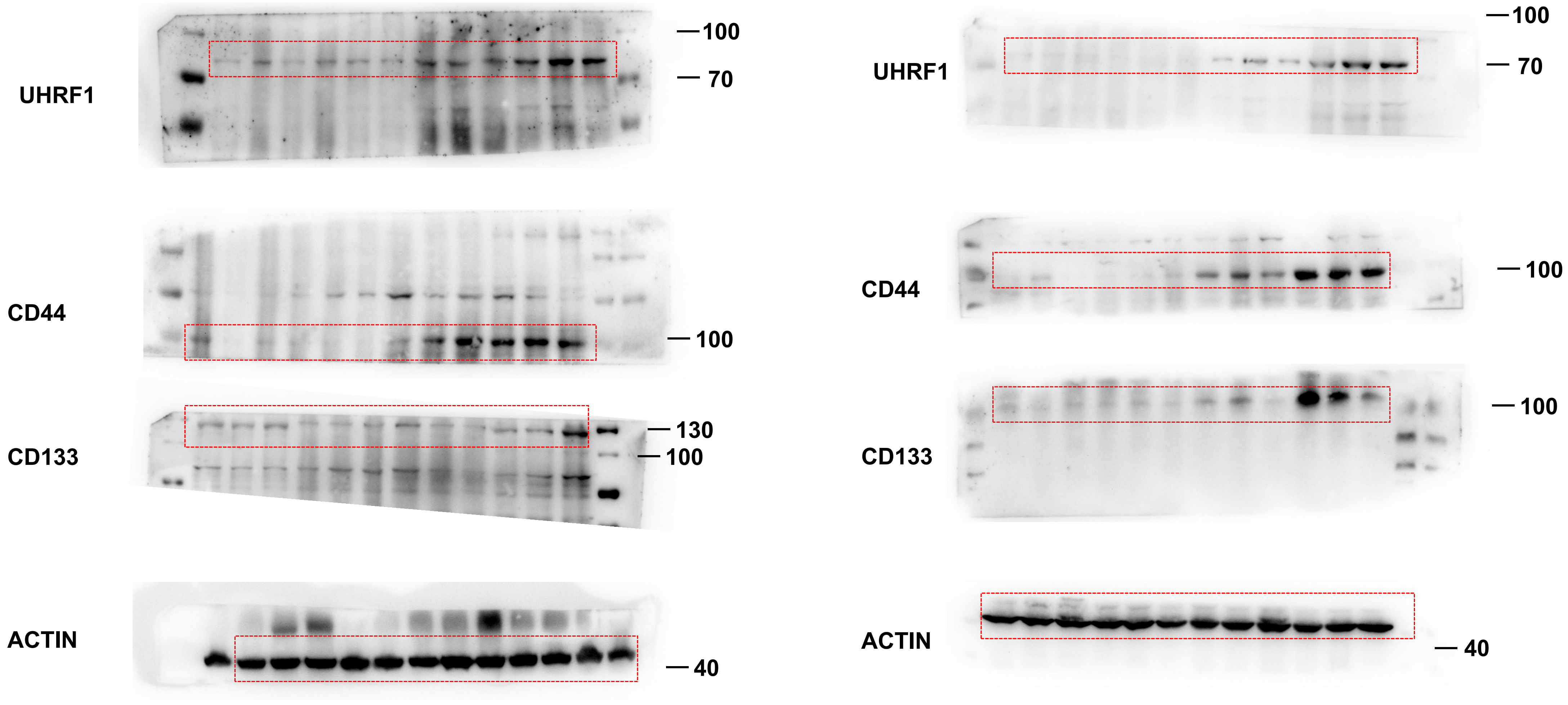

Fig S2C (original blots)

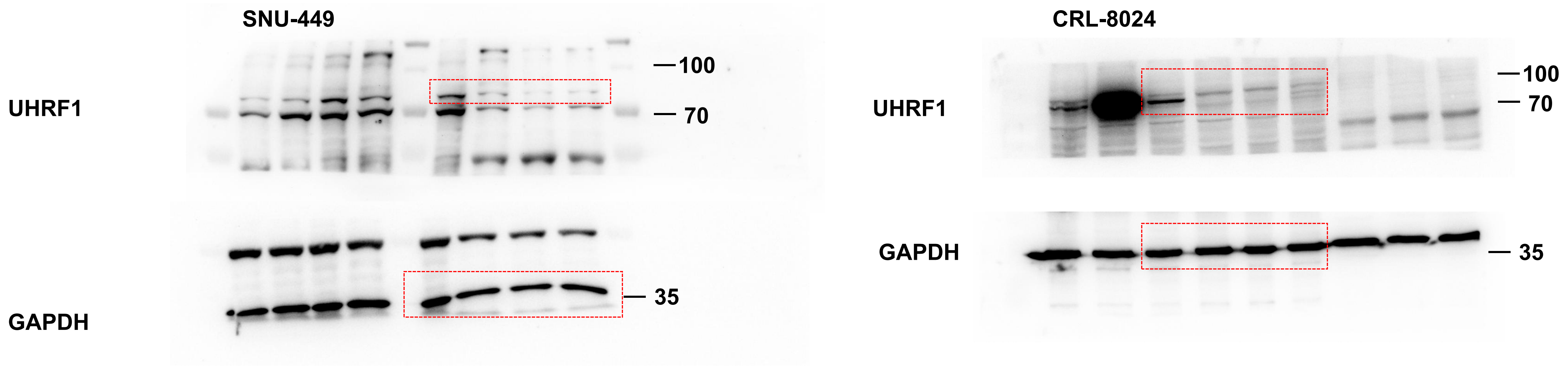

Fig S3A (original blots)

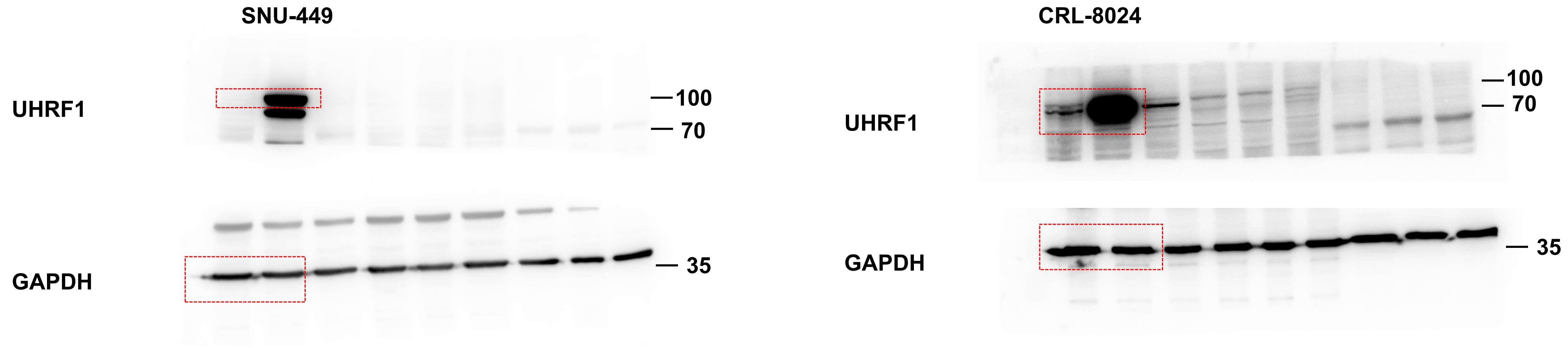

Fig 4D (original blots)

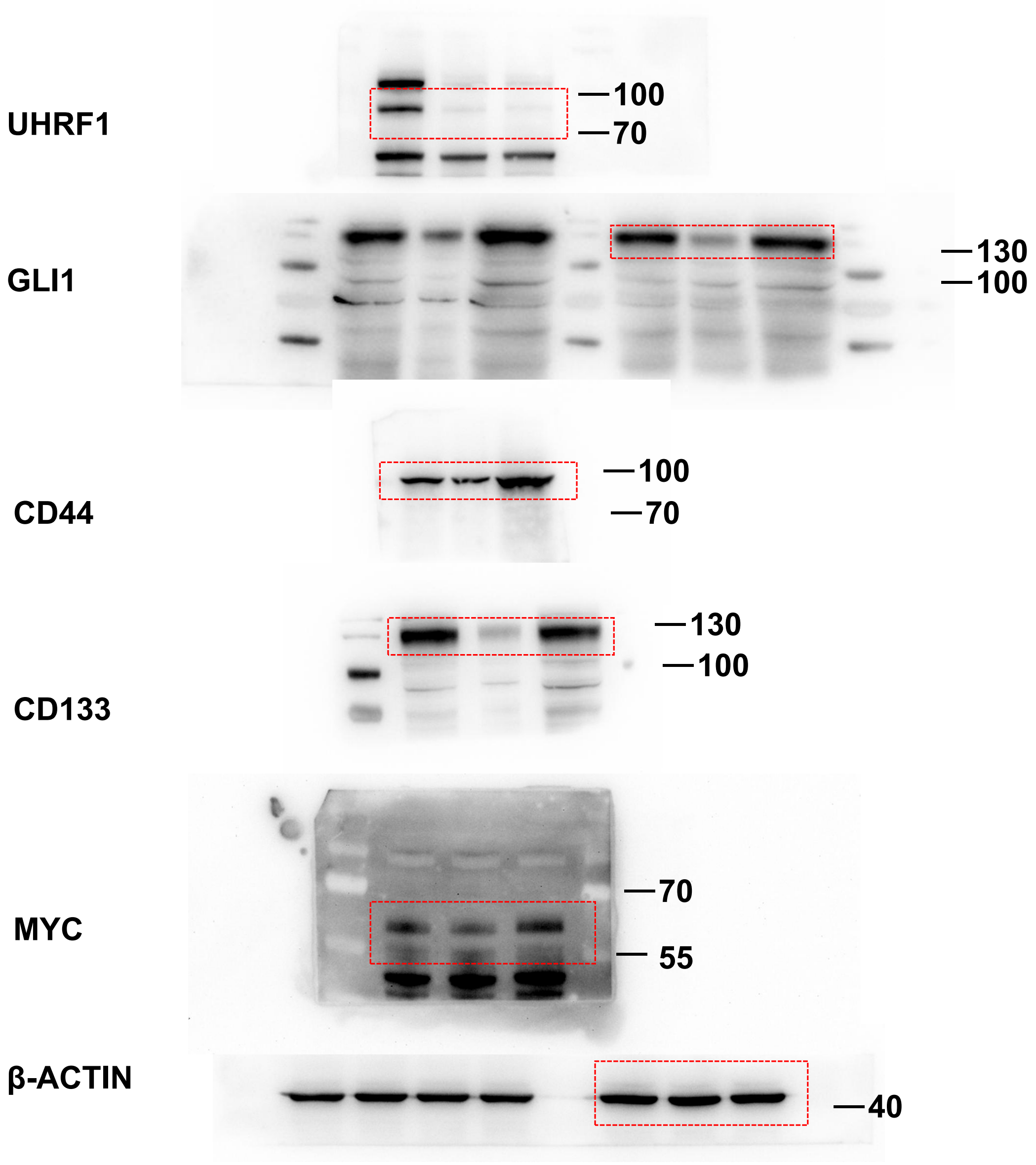

Fig 5B (original blots)

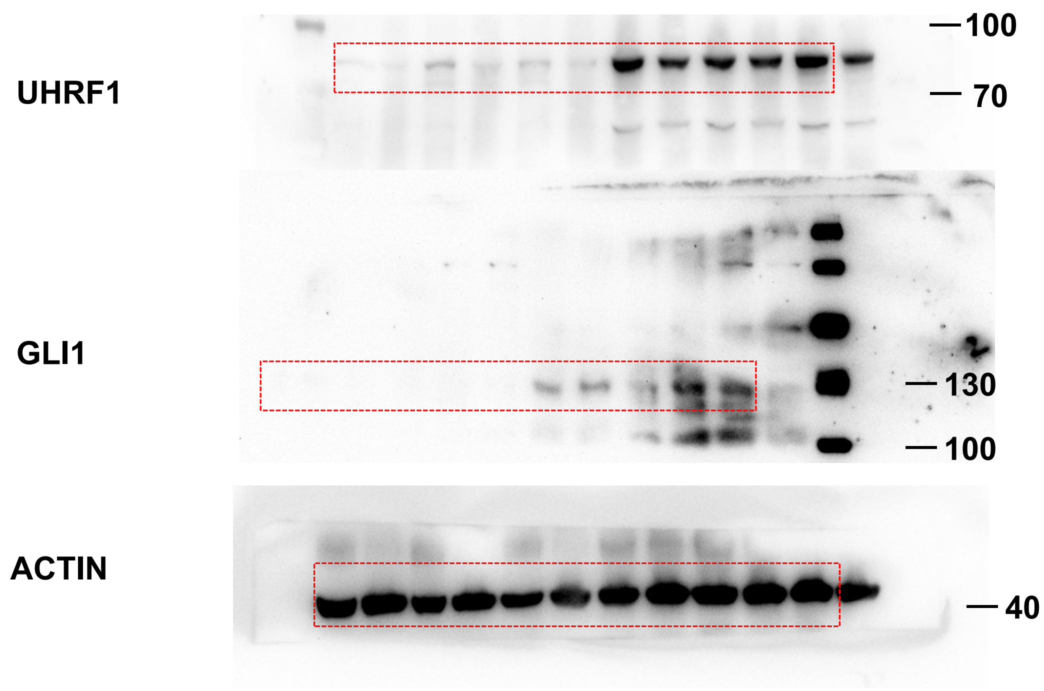

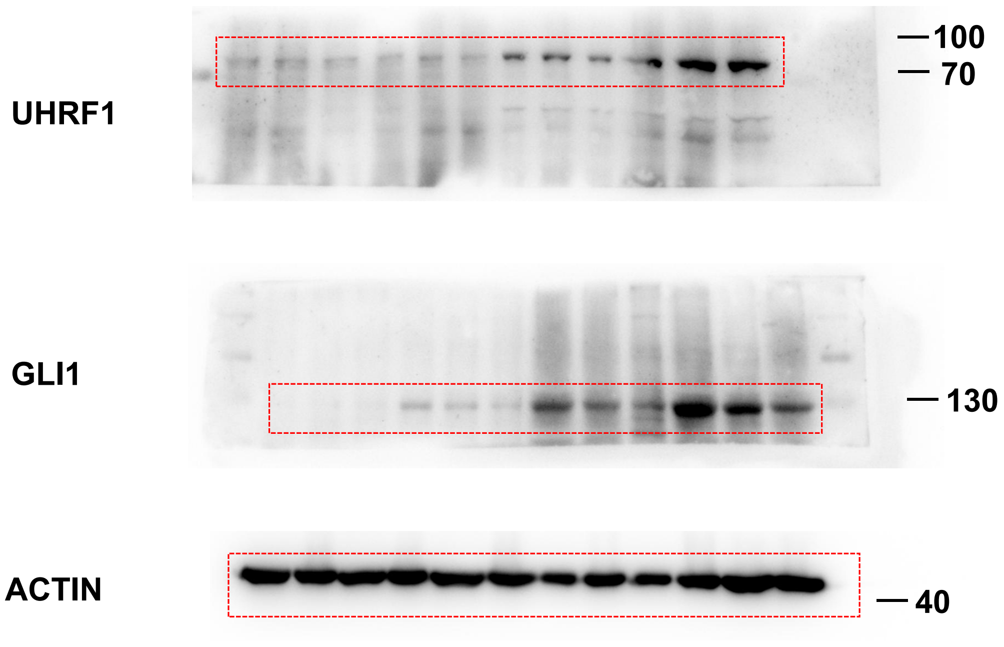

Fig S6B (original blots)

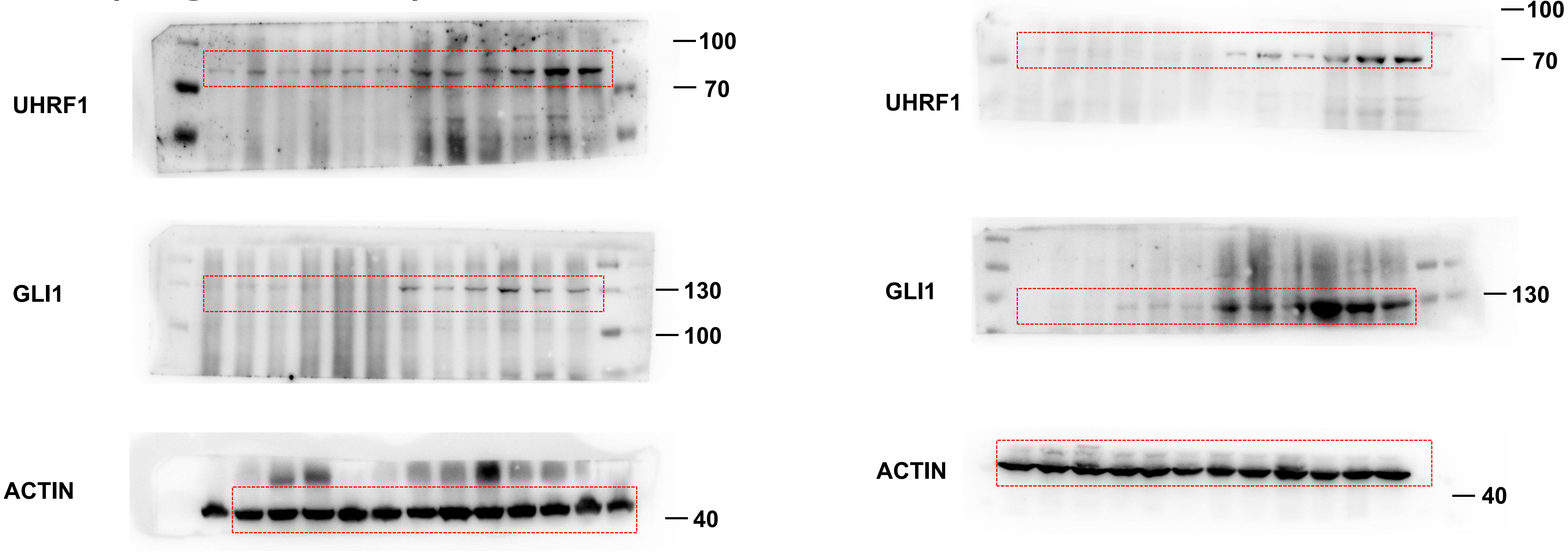

Fig 6E (original blots)

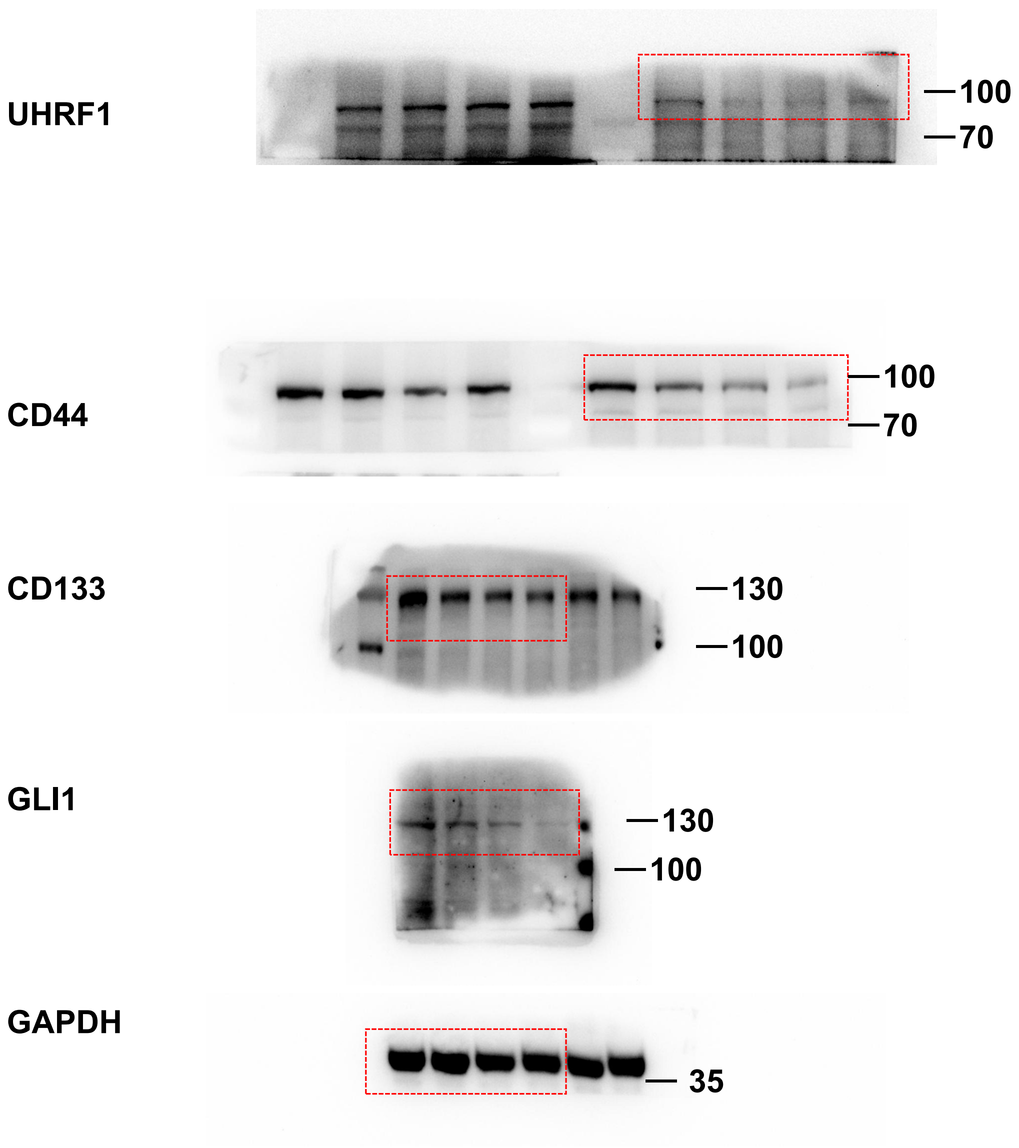

Fig 6H (original blots)

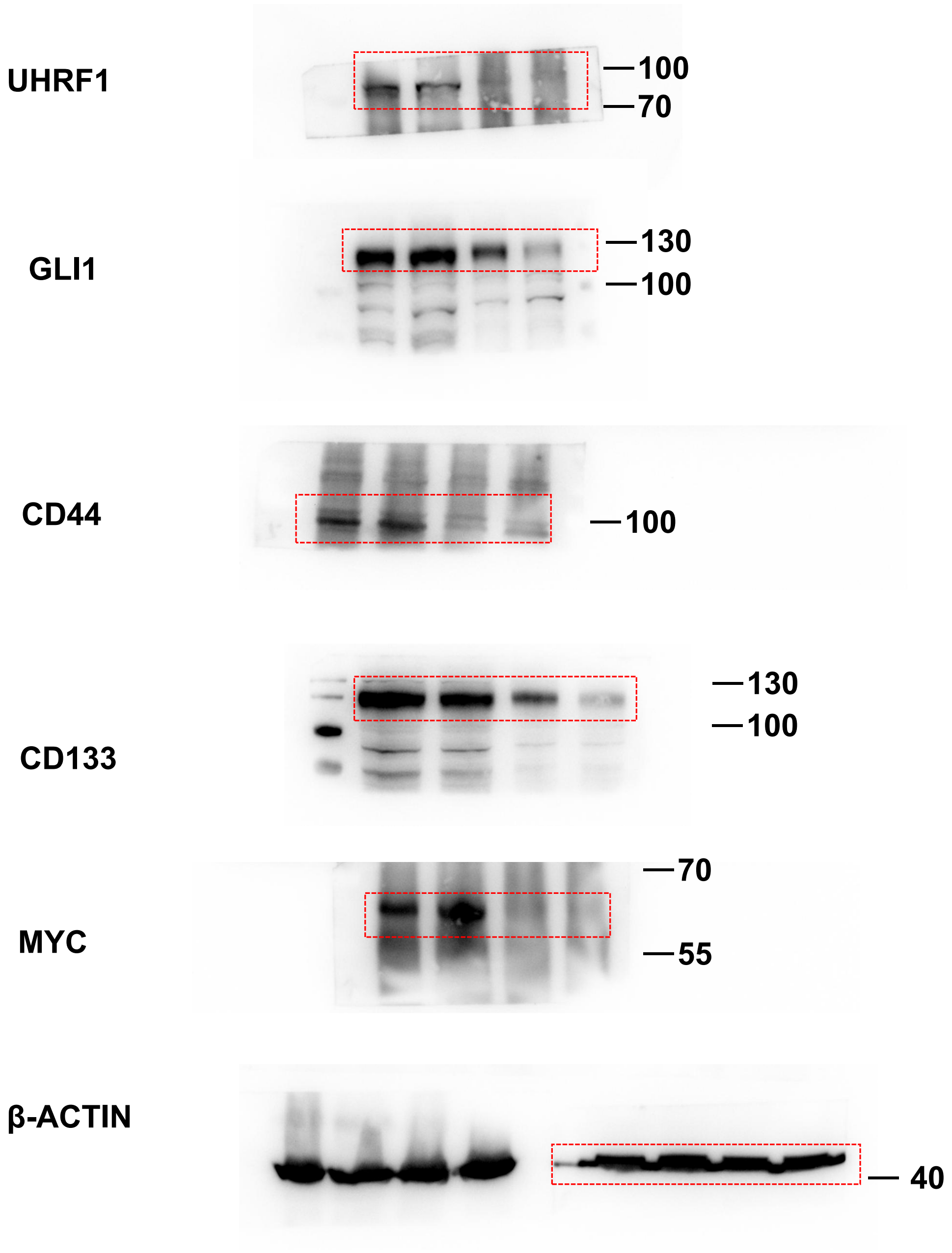

Fig 7F (original blots)

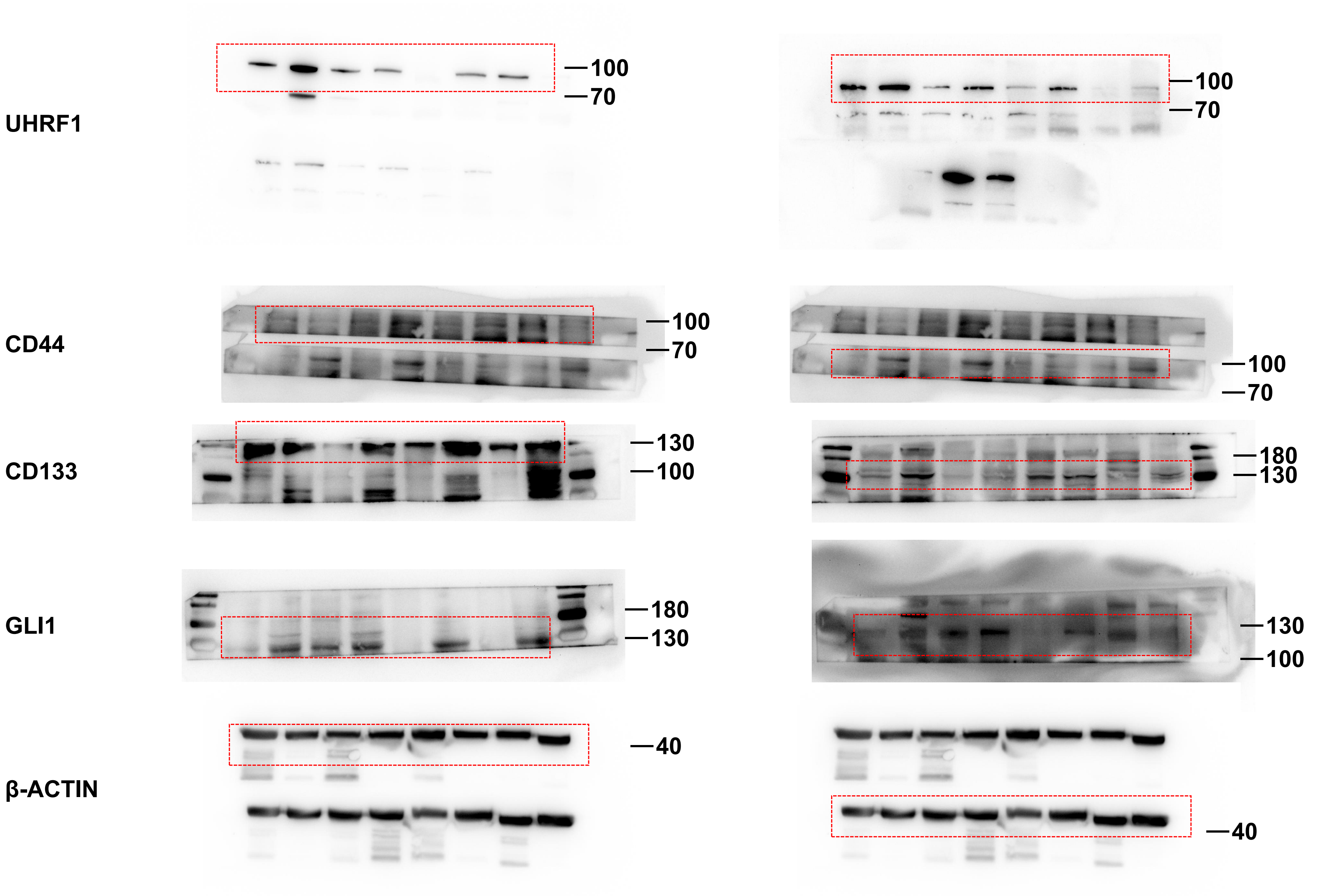

Supplement: Supplementary file 3 — Supplemental Material - Original Blots [file 41419_2023_5895_MOESM3_ESM.pdf]
